# Cryo-EM reveals the quaternary architecture of TenpIN type III toxin-antitoxin RNP complex common to pathogenic bacteria

**DOI:** 10.1371/journal.ppat.1014690

**Published:** 2026-10-06

**Authors:** Kavyashree Nadig, Surekha Padmanaban, Salik Noor, Sankaran Sandhya, Parthasarathy Manikandan, Somnath Dutta, Mahavir Singh

**Affiliations:** Molecular Biophysics Unit, Indian Institute of Science, Bengaluru, Karnataka, India; National Jewish Health, UNITED STATES OF AMERICA

## Abstract

Type III toxin-antitoxin (TA) systems are anti-phage defense systems in bacteria that comprises a protein toxin and a cognate non-coding RNA antitoxin. Under normal conditions, the antitoxin binds and inhibits the toxin. Upon phage infection, antitoxin is neutralized, releasing the active toxin, which cleaves the bacterial as well as phage RNA and halts viral replication. These systems are classified into *tenpIN*, *cptIN*, and *toxIN* families based on toxin sequence homology. While the ToxIN and CptIN RNP complexes have been structurally characterized, TenpIN systems remained poorly understood for their structure and assembly. Here, we report the functional and structural characterization of a TenpIN system found in several pathogenic bacteria. The antitoxin RNA, *tenpI*, contains two non-identical repeats, both capable of binding TenpN toxin, and rescuing toxin-induced growth inhibition in *E. coli*. Using single-particle cryo-electron microscopy and *de novo* modeling, we determined the structure of the TenpIN complex as a closed hetero-tetramer comprising two TenpN proteins and two *tenpI* RNAs. The antitoxin RNA folds into a central pseudoknot flanked by single-stranded regions interacting with the toxin. Structural analysis revealed a close resemblance between TenpN and ToxN, while *tenpI* RNA shares structural features with *cptI* RNA, thereby expanding our understanding of the structural diversity of Type III TA systems.

## Author summary

Bacteria have evolved sophisticated mechanisms to survive stressful conditions and defend themselves against invading bacteriophages. Toxin-antitoxin (TA) systems are one such defence mechanism. They consist of a toxin that can inhibit bacterial growth and an antitoxin that keeps the toxin inactive under normal conditions. In type III TA systems, the antitoxin is an RNA molecule that directly binds and neutralizes the protein toxin. Although several type III TA systems have been studied, the *tenpIN* family remains poorly understood in terms of its structure and assembly. Recent bioinformatic analyses have revealed that *tenpIN* systems are much more widespread than previously recognized, including in important antibiotic-resistant bacterial pathogens, highlighting the importance of understanding their molecular mechanisms. Here, we report the functional characterization, purification, and quaternary structure of a TenpIN ribonucleoprotein complex that is conserved across several pathogenic bacteria. The structure revealed that the TenpIN complex contains two toxin proteins (TenpN) and two antitoxin RNA (*tenpI* repeats) molecules. We have also identified and experimentally validated putative active-site residues of TenpN. These findings expand our understanding of the structural and functional diversity of type III TA systems and provide a foundation for exploring their roles in bacterial defence, biotechnology, and antimicrobial research.

## Introduction

Toxin-antitoxin (TA) systems are widespread among bacteria and archaea, where they play roles in stress response, plasmid maintenance, and phage defence [1,2]. In general, TA systems are composed of a toxin (usually a protein) and its cognate antitoxin (which can be RNA or protein) [3,4]. Under normal growth conditions, the toxin is kept inactive by the antitoxin. However, during stress conditions, this regulation is lost, leading to the activation of toxin that has growth inhibitory effects on the host bacteria. In type III TA systems, an RNA antitoxin neutralizes the protein toxin [5]. Type III TA systems have been classified into three families: *toxIN*, *cptIN*, and *tenpIN* families [6,7]. Among them, the *tenpIN* family remains one of the least studied for their structure, assembly, and functions. First identified over a decade ago, around 25 putative *tenpIN* sequences were reported and classified into various families based on sequence similarities [6]. A TenpIN system, identified in *Photorhabdus luminescens* TT01, was the first TenpIN system functionally characterized in a heterologous host, *E. coli* [8]. This system was found to act as a functional type III TA system, where its RNA antitoxin was shown to neutralize the toxicity of the TenpN protein toxin [6]. Furthermore, the full *tenpIN* locus from *P. luminescens* provided resistance to phage infections, particularly against environmentally isolated coliphages such as ϕ_TB27, ϕ_TB28, and ϕ_F6 [8]. However, the phage resistance profile of *tenpIN* differed from the well-studied *toxIN* system, where other phages such as ϕ_S61 and ϕ_TE were aborted by *toxIN* but not by *tenpIN*. Additionally, M1-23 mutation in phage ϕM1, allowed its escape from both *toxIN*_*Pa*_ and *tenpIN*_*Pl*_ systems, highlighting the complexity of the arms race between phages and bacterial TA systems [8]. In another study, T4-like phages were isolated from sewage that were initially sensitive to *toxIN*_*Pa*_ but evolved resistance through mutations in *asiA* and *orf84* genes, or by deleting a large genomic region, providing insight into phage factors involved in *toxIN*_*Pa*_ activation [9].

Despite the early work on *tenpIN* from *P. luminescens*, no other putative *tenpIN* systems have been functionally characterized to date. Subsequently, *tenpIN* systems were identified in three different strains of *Staphylococcus aureus* [10] and recently over 700 unique *tenpN* sequences across prokaryotic genomes and viruses were identified in a bioinformatic study, suggesting that *tenpIN* systems are far more widespread than were previously reported [11]. Some of these newly identified systems feature unconventional antitoxin repeats such as non-identical repeats, posing interesting questions for their structure and binding to the toxins. Moreover, several *tenpIN* systems were discovered in ESKAPE pathogens, which are responsible for most of the antibiotic-resistant infections [12]. This raises the potential for *tenpIN* systems to be exploited as novel antibacterial targets [13,14].

The *toxIN* and *cptIN* families have been studied for their structures and assembly [15–18]. The ToxIN TA systems form a cyclic hetero-hexameric RNP complex [16–19], whereas the CptIN forms a hetero-tetrameric RNP complex assembly [15]. In this study, we report the structural characterization of the first TenpIN TA complex, using cryo-electron microscopy (cryo-EM). Interestingly in this system the antitoxin RNA consists of two non-identical repeats. We show that both the repeats can potentially form pseudoknot structures and neutralize the toxin. The structure reveals that TenpIN complex forms a hetero-tetrameric RNP assembly similar to the CptIN system, consisting of two toxin proteins and two antitoxin RNA molecules. The structure also reveals putative active site of the TenpN toxin endoribonuclease that was validated using a mutagenesis approach.

These results broaden our understanding of type III TA systems and highlight the diversity within *tenpIN* class. The study also opens new avenues for exploring the evolutionary relationships, functional mechanisms, and potential applications of type III TA systems in bacterial defence and biotechnology.

## Results

### A putative TenpIN system common to several pathogens is a functional type III TA system

In this study, we selected a putative *tenpIN* system which was common to several pathogens (where toxin TenpN shared 100% sequence identity) that include *Salmonella enterica, Shigella flexineri, Klebsiella pneumoniae,* and *Escherichia coli*, to study its structure and functions (S1 Fig) [11]. A typical type III TA operon includes a common promoter, antitoxin repeats, a rho-independent hairpin terminator followed by the toxin encoding sequence. The genetic organization of the putative *tenpIN* TA system under study is shown in Fig 1a. The *tenpIN* operon contains an antitoxin gene encoding a non-coding RNA antitoxin cassette of 102 nucleotides and a toxin gene encoding a protein toxin of 153 amino acids residues. There is a putative Rho-independent terminator sequence between the antitoxin and the toxin genes (Fig 1a).

**Fig 1:**
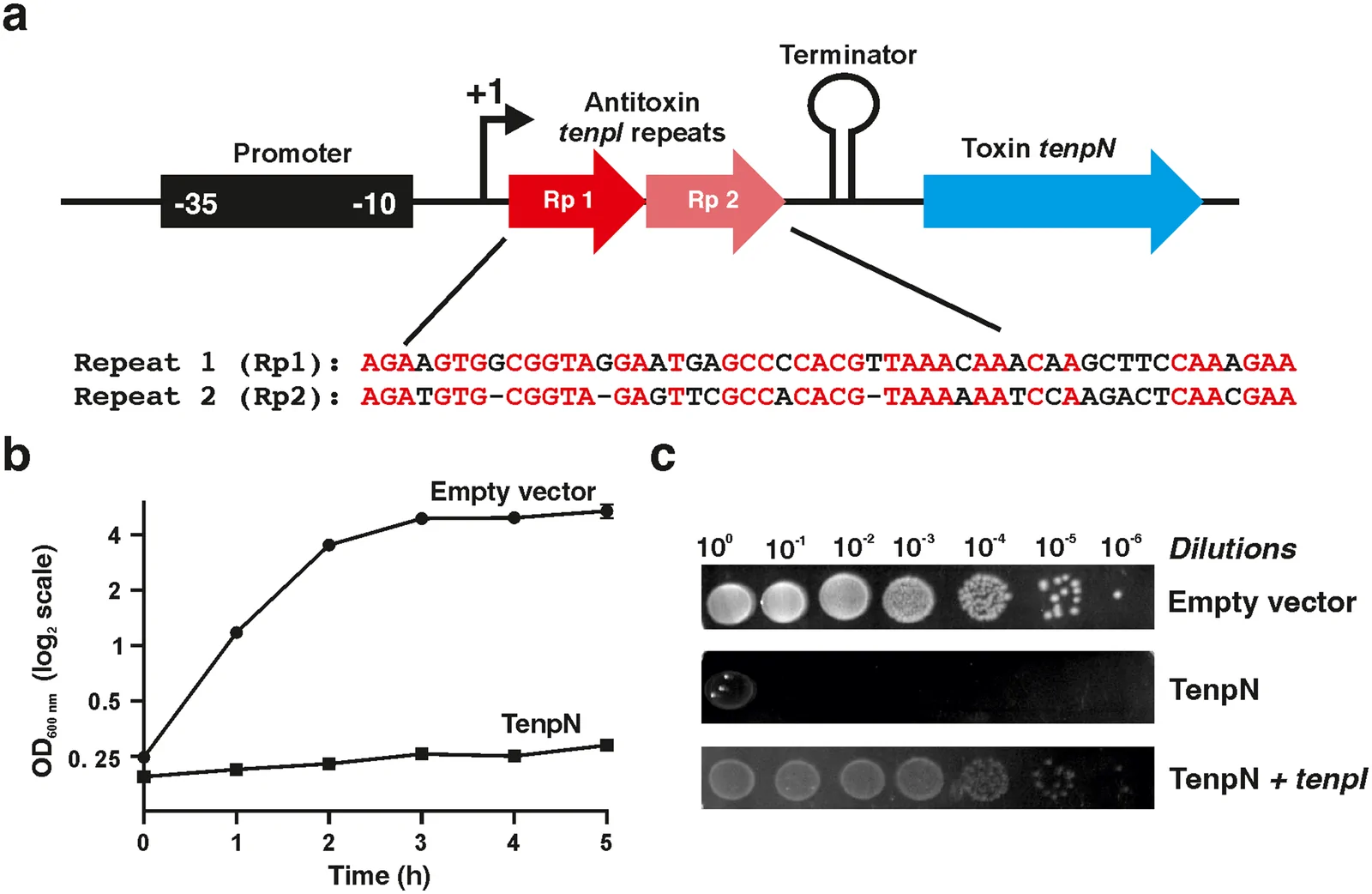
A t*enpIN* system common to several pathogenic bacteria is a functional type III TA system. **a)** Operon structure of *tenpIN* system studied under current study. The antitoxin *tenpI* encodes two non-identical repeats. Sequence alignment of both the repeats is shown. **b)** *E. coli* growth curve showing the bacterial growth inhibition upon expression of toxin TenpN. **c)** *E. coli* spot growth assays shows that TenpN induced growth inhibition is rescued by expression of *tenpI* antitoxin RNA.

To assess the functionality of this system, we used *E. coli* growth assays. The *tenpN* toxin gene was over expressed in *E. coli MG1655* cells. The *E. coli* growth upon expression of TenpN toxin showed a significant bacterial growth inhibition compared to the empty vector control (Fig 1b). We observed similar results on a solid media by spotting 10-fold serial dilutions of cultures on LB agar plates (Fig 1c). The toxin induced bacterial growth inhibition was rescued when the putative *tenpI* encoding sequence was co-expressed in *trans* (Fig 1c). These results confirm that the putative sequence encodes a functional *tenpIN* TA system and TenpN toxin is bacteriostatic in nature.

### *tenpI* antitoxin consists of two non-identical, functional repeats

The antitoxins of type III TA systems (except for the non-canonical avcID system) [20] contain multiple tandem antitoxin repeat sequences [7]. Toxin gene encodes a specific endoribonuclease that processes the full-length antitoxin repeats into the functional repeats. Functional repeats in turn inactivate the toxin by forming a TA RNP complex. In the previously reported *toxIN* and *cptIN* systems, the antitoxin repeats are near identical (differing in 1–2 nucleotide positions throughout the entire repeat length) [6,15,17,21]. In contrast, in a large subset of *tenpIN* systems, the antitoxin repeats exhibit multiple mismatches relative to one another, making them non-identical at the sequence level (Nadig et al., 2025). In type III TA systems consisting of identical antitoxin repeats (*toxIN* and *cptIN*), previous studies have demonstrated that a single complete functional RNA antitoxin repeat is sufficient to inhibit toxin activity [6,21].

In case of the *tenpIN* system characterized in this study, the tandem repeat finder [22] could not identify any RNA repeats in the putative antitoxin coding gene (region between promoter and terminator sequence). When the sequence was manually inspected, two sequences that were similar to each other but not identical were identified (Fig 1a). These two repeats were termed as putative antitoxin repeat 1 and 2 (Rp1 and Rp2 respectively). The alignment of Rp1 and Rp2 sequences showed that although the positions of the stems and loops that would form the pseudoknots were conserved, several other nucleotide positions are different in both the repeats (Fig 2a). Further, Rp2 is shorter by three nucleotides as compared to the Rp1 (Fig 1a and Fig 2a and 2b).

**Fig 2:**
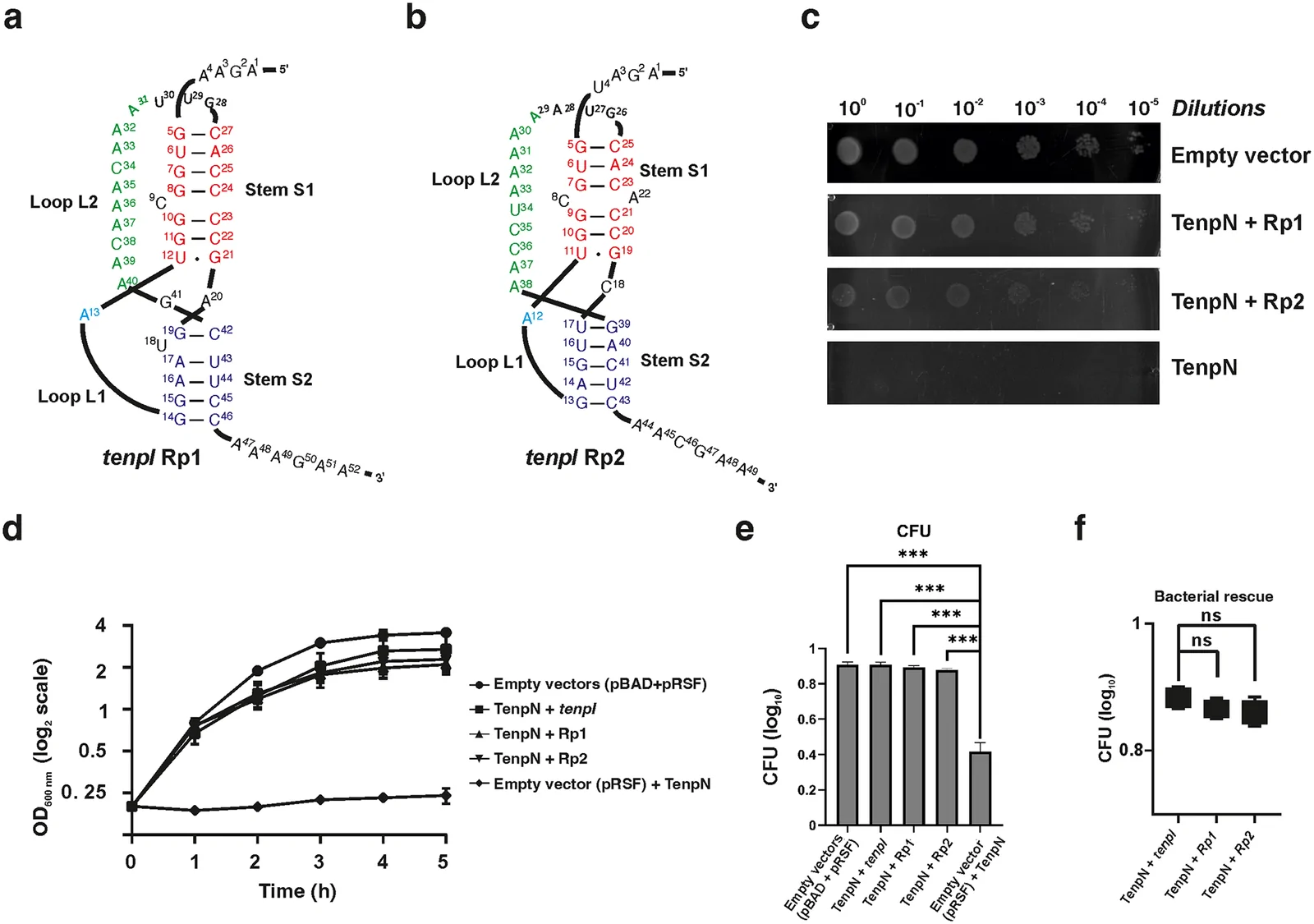
Antitoxin *tenpI* repeats rescue bacteria from TenpN mediated growth inhibition. **a)** Predicted secondary structure of repeat 1 (Rp1) of *tenpI* antitoxin. **b)** Predicted secondary structure of repeat 2 (Rp2) of *tenpI* antitoxin. **c)** *E. coli* spot growth assays showing that TenpN induced growth inhibition is rescued by both Rp1 and Rp2. **d)** *E. coli* growth curves showing growth inhibition upon TenpN expression and its rescue by Rp1, Rp2 or full length *tenpI* independently. **e)** Colony forming units (CFU) assays showing that both Rp1 and Rp2 rescue similar number of bacterial colonies. **f)** Toxin rescue assay demonstrating comparable CFUs for full-length *tenpI*, Rp1, and Rp2, indicating that all constructs similarly counteract TenpN-induced growth inhibition.

Structures of previously characterized Type III TA systems (i.e., ToxIN and CptIN RNP complexes) showed that the antitoxin repeats are well structured and consist of a central H-type pseudoknot. Therefore, we inspected the *tenpI* Rp1 and Rp2 sequences for their ability to form potential H-type pseudoknots. Interestingly, both the sequences showed potential to form H-type pseudoknot (Fig 2a and 2b).

Previous studies on the *toxIN* systems, had shown that small changes (e.g., single point mutation) in antitoxin repeat rendered it inactive to bind and inactivate the toxin [15,16,21,23]. This prompted us to check whether both Rp1 and Rp2 can act as functional antitoxin repeats and inhibit the TenpN toxin’s activity. To test this, Rp1 and Rp2 were cloned separately and co-transformed with TenpN plasmid in *E. coli* to rescue the toxin-induced growth inhibition. Interestingly, both Rp1 and Rp2 were able to restore bacterial growth when co-expressed with TenpN as monitored by bacterial growth assays (Fig 2c and 2d). Spot-growth assays on solid LB agar media (Fig 2c) showed bacterial rescue by both Rp1 and Rp2 individually, though we observed less dense colonies in case of Rp2 rescue at all the dilution points (Fig 2c). To corroborate this, we performed colony forming unit (CFU) experiments that revealed that co-expression of TenpN with *tenpI* full length (Rp1 + Rp2), Rp1, or Rp2 leads to significant increase in CFU of *E. coli* cells compared to the expression of TenpN alone (Fig 2e).

Furthermore, most type III toxins are known to have a bacteriostatic effect even after expressing for several hours [17,19,21]. To assess which antitoxin RNA repeat most effectively rescues bacterial cells from TenpN toxicity, we performed toxin rescue assays in which TenpN was overexpressed for 2.5 h together with Rp1, Rp2, or *tenpI* full length, and cell viability was measured under TenpN-suppressed conditions (Fig 2f). Both Rp1 and Rp2 were able to rescue similar number of bacterial cells indicating that both the repeats were functional to a similar extent.

### TenpIN complex forms a heterotetrameric assembly

The TenpIN complex was expressed and purified as described in the Material and Methods section. In summary, the toxin and antitoxin gene containing plasmids were co-transformed, co-expressed, and co-purified using a strategy that had previously been standardized [24] (S2a-S2e Fig). Presence of TenpN toxin protein and *tenpI* RNA repeats copurified after His-tag Ni^2+^-NTA affinity chromatography was confirmed using denaturing SDS-PAGE (S2a Fig) and urea-PAGE (S2b Fig). At the ion-exchange chromatography step, TenpIN complex and a small amount of TenpN were eluted separately at different salt concentration along the gradient (S2c Fig) and purified further on a size-exclusion chromatography column. The TenpIN complex eluted at an approximate molecular weight size of ~72 kDa from a size-exclusion chromatography column, that would correspond to a heterotetrameric assembly consisting of two TenpN toxins and two *tenpI* antitoxin molecules (S2d Fig). Free TenpN eluted as a monomer from the size-exclusion chromatography column (S2e Fig).

We looked for the presence of Rp1 and Rp2 antitoxin repeats in the eluted TenpIN complex. For this, sequences corresponding to the Rp1 and Rp2 RNAs were reversed transcribed and the cDNAs were amplified and sequenced using Sanger’s sequencing method. Sequencing results showed the presence of both Rp1 and Rp2 in the purified TenpIN complex (see Material and Methods section).

Next, we posed the questions if Rp1 and Rp2 form stable structures and whether they can assemble with TenpN toxin independently *in vitro*. The Rp1 and Rp2 RNAs were *in vitro* transcribed and purified (S2f Fig). To test stabilities of Rp1 and Rp2 structures, we used CD spectroscopy based thermal melting experiments (S3a and S3b Fig). The results showed that both Rp1 and Rp2 unfold with melting temperature (Tm) of ~56 ºC, showing that both the repeats are thermally stable to the same extent (S3c Fig).

The purified TenpN was incubated with the *in vitro* transcribed and purified Rp1 and Rp2 antitoxin repeats of the *tenpI* RNA, and the complex was run on an analytical size-exclusion chromatography column (S4 Fig). Both TenpN-Rp1 and TenpN-Rp2 complexes eluted at higher molecular weight (compared to the free TenpN), proving complex formation in both the cases (S4 Fig).

### TenpIN TA RNP complex displays inherent C2 symmetry

The purified TenpIN complex was analyzed through negative-stain transmission electron microscopy (TEM). The TEM images of the fractions from the left slope of the size-exclusion chromatography (SEC) elution peak showed homogeneous particles (S5 Fig), while those from the centre (S6a Fig) or right (S6b Fig) slope of the SEC peak fractions showed broken or heterogeneous particles. Therefore, fractions from the left slope were considered for further analysis. The negative staining TEM data was processed to generate 2D class averages from 6000 particles to further confirm the orderedness and stability of the complex (S5 Fig).

After standardizing TenpIN TA particles for homogeneity and good concentration using TEM, we proceeded with cryo-EM data collection and processing (Fig 3a and 3b and S7 Fig). The initial 3D reconstruction from the extracted particle images identified densities corresponding to protein and RNA, revealing that the TenpIN TA complex forms a closed cyclic hetero-tetrameric assembly of two protein and two RNA moieties (Fig 3c). The observed 2:2 protein to RNA stoichiometry of TenpIN matches with the analytical SEC result that had shown that TenpIN complex elutes as an apparent heterotetrameric complex (S2d Fig).

**Fig 3:**
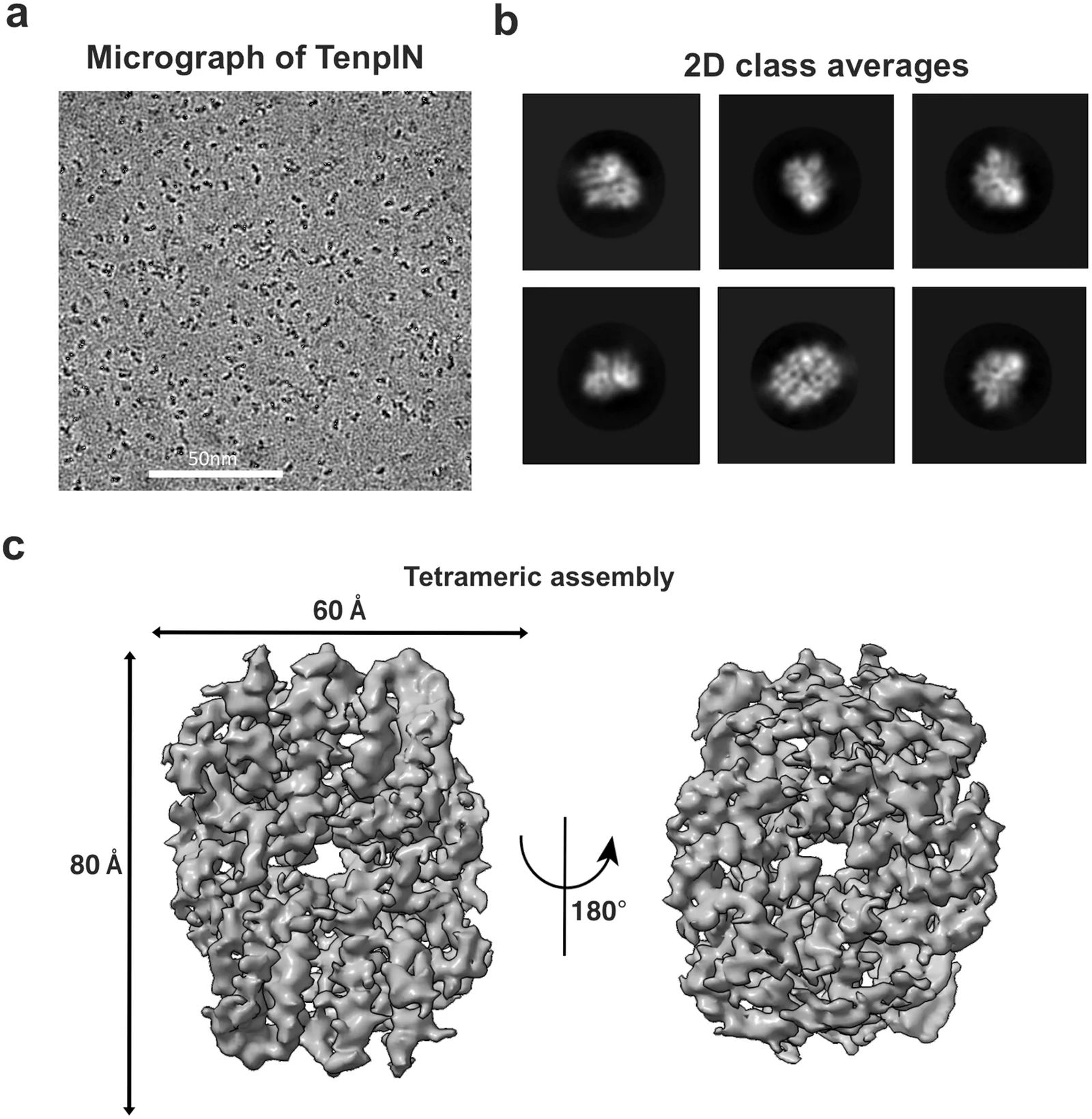
Cryo-EM single-particle analysis of TenpIN complex. **a)** Raw Cryo-EM micrograph of TenpIN complex showing homogenous particles. **b)** Representative 2D class averages show features of TenpIN complex. **c)** 3D reconstruction of *E. coli* TenpIN complex of approximate dimension of 80 Å X 60 Å with a C2 symmetry.

542913 particles were classified using C2 symmetry to obtain 3D class averages (S7 Fig). Classified map with good features was used for the final 3D refinement, using 327240 particles to achieve a resolution of ~ 3.9 Å (S7 Fig). The resulting symmetric (C2) and asymmetric maps (C1) were almost identical, indicating no significant structural differences. Angular distribution (S7 Fig) showed the distribution of particles in various orientations, demonstrating no orientational bias.

### Cryo-EM revealed the quaternary structure of TenpIN RNP complex comprising a hetero-tetrameric assembly

The cryo-EM structure of TenpIN TA complex was elucidated in its native state, without any antibody or additional tags. Despite the complex’s relatively small size (~72 kDa for the hetero-tetrameric assembly), the sharpened map clearly distinguished two TenpN proteins and two TenpI RNA molecules (Fig 4a). The overall dimensions of TenpIN complex was approximately 80 Å in length and 60 Å in width (Fig 3c). The RNA and protein are arranged alternately, with single stranded RNA density from two different RNA molecules coming close together on the same surface of the protein toxin (Fig 4a). Supplementary S7 Fig illustrates the local resolution of the TenpIN density map, indicating that the highest resolution is achieved at the core of the complex and it gradually diminishes towards the periphery. From the density map, it could be observed that the TA complex indeed has a C2 symmetry with a hole at the centre suggesting that the proteins protomers do not interact with each other to make the hetero-tetrameric structure.

**Fig 4:**
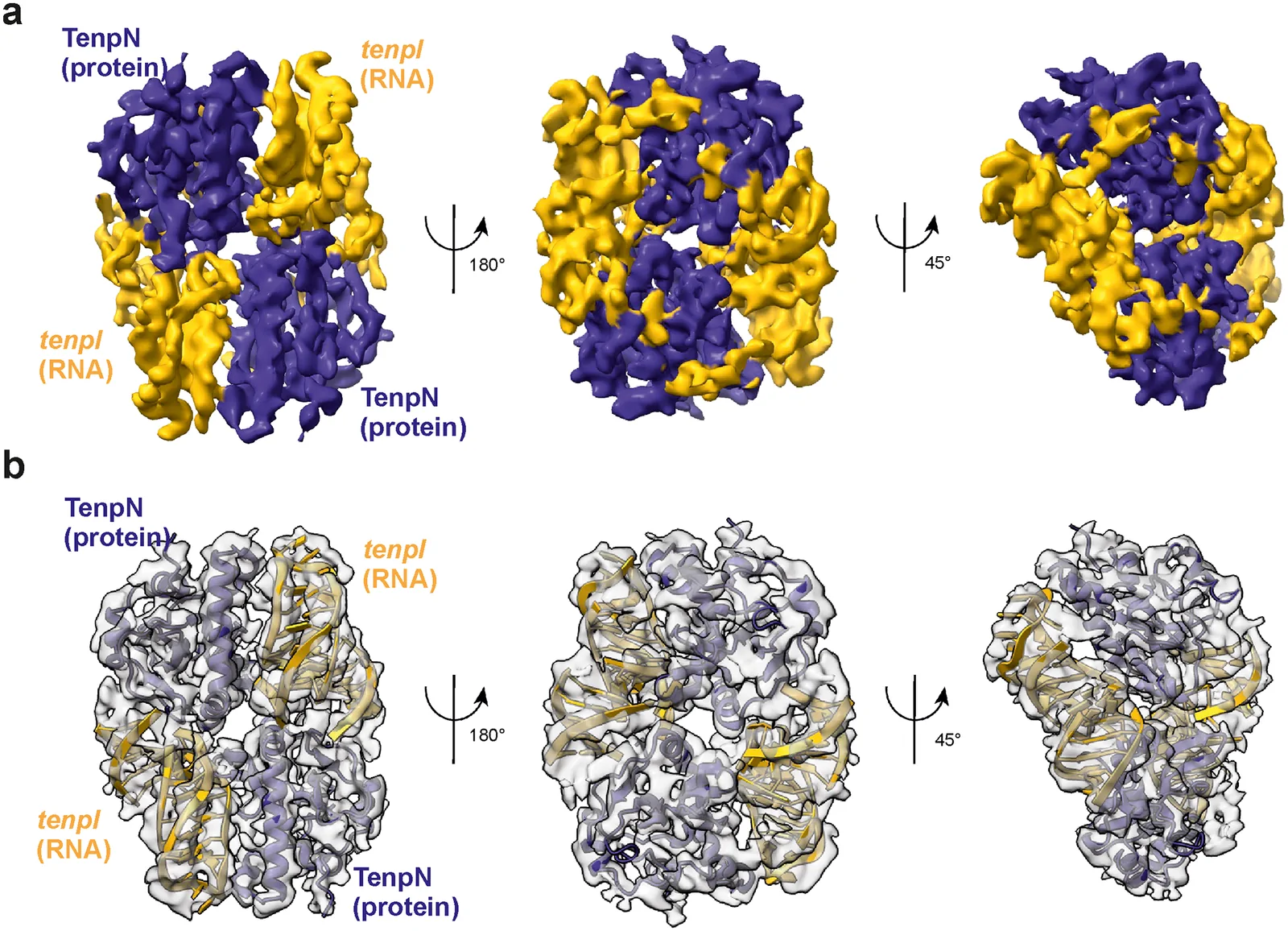
Cryo-EM reveals the quaternary structure of TenpIN complex. **a)** Cryo-EM densities reveal that one TenpIN complex contains two copies of TenpN protein toxin (in blue) and two copies of *tenpI* RNA antitoxin (in yellow). **b)** TenpN protein and *tenpI* RNA models fitted into the refined cryo-EM densities. Protein and RNA models are shown in blue and yellow colours respectively.

The initial TenpN protein model was generated using AlphaFold2 [25]. This model was then fitted and refined into the experimentally obtained cryo-EM density (Fig 4b). Building the RNA tertiary structure model from its primary sequence required multiple approaches (see Material and Methods). Briefly, the RNA models, for Rp1 and Rp2 were constructed fragment by fragment and then assembled in Coot [26], Chimera [27] and refined locally in Phenix [28]. Various tools were utilized to identify stems of the pseudoknot, with the core pseudoknot modelled using the simRNA tool [29]. Finally, RNA and proteins were fitted into the cryo-EM density, (Fig 4b and Fig 5a-5f).

**Fig 5:**
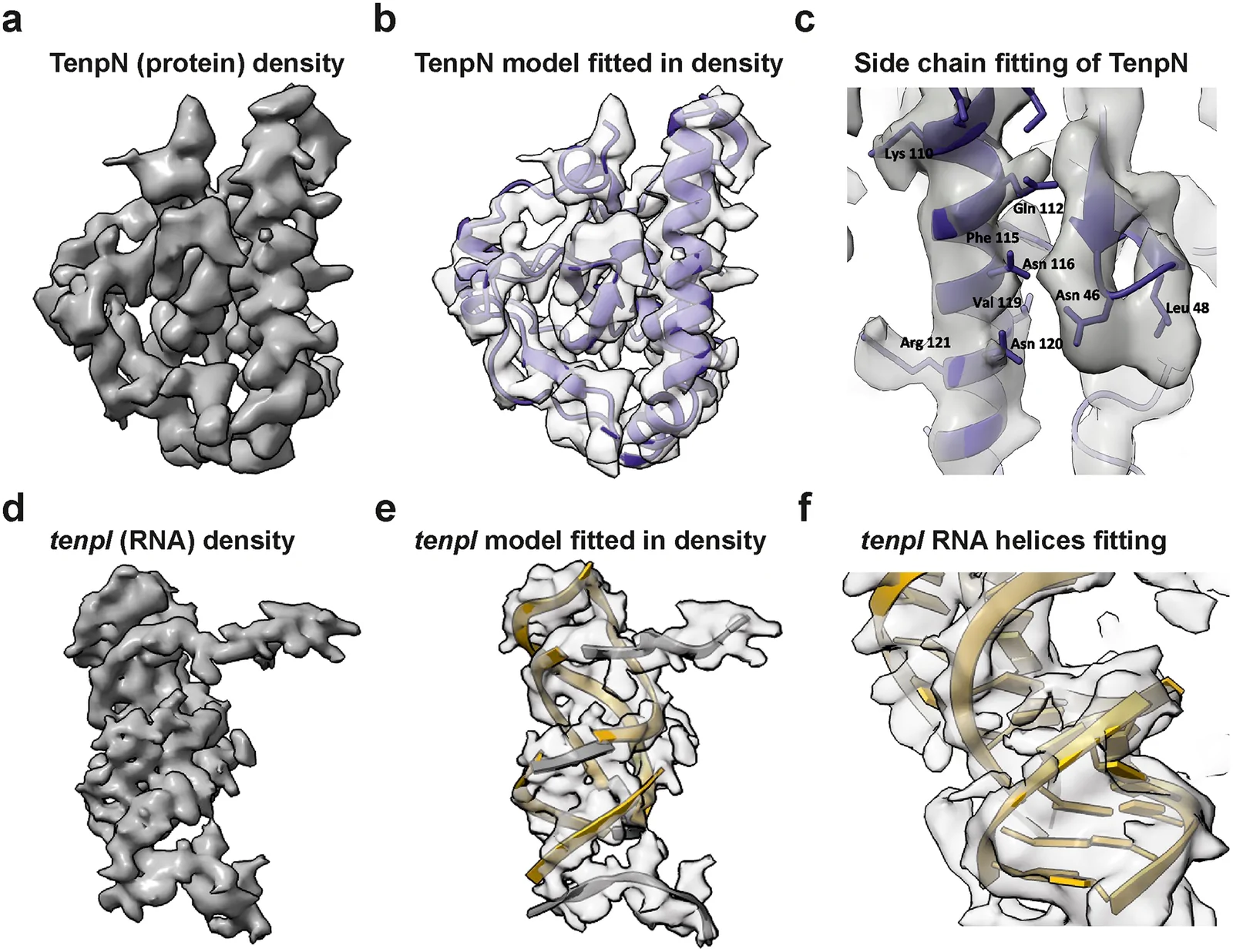
Cryo-EM densities and fitted models of TenpN toxin and *tenpI* RNA. **a)** Refined cryo-EM density of TenpN toxin segmented from TenpIN map. **b)** TenpN toxin model fitted into the refined cryo-EM density. **c)** Snapshot of a region of TenpN toxin showing the fitting of the side chains of some of the amino acid residues. **d)** Refined cryo-EM density of *tenpI* RNA repeat segmented from TenpIN map. **e)** Rp1 *tenpI* antitoxin model fitted into the refined cryo-EM density. **f)** Snapshot of region of Rp1 *tenpI* RNA clearly showing the A-form of RNA helices.

Rp1 model exhibited a more accurate overall fit within the cryo-EM density map compared to Rp2. The shorter length of Rp2, which lacks three nucleotides relative to Rp1, may contribute to the comparatively poorer fit to the observed TenpIN cryo-EM density. This was also reflected in the map-to-model Correlation coefficient values (CC volume), which reflects the fitting of the atomic model to the map and is hence used to validate cryo-EM structures, of Rp1 and Rp2 fitting. Consistent with the visual assessment, we obtained better CC volume value for Rp1 (CC volume 0.66) fitting than Rp2 (CC volume 0.52) fitting (S8a and S8b Fig). Therefore, we selected Rp1 for the final fitting into the cryo-EM density map and for the representation of the TenpIN complex in this manuscript.

Overall, the protein model fitted well within the protein density of the cryo-EM map, displaying a good fit even for the amino acid side chains (Fig 5a-5c). The two protein protomers in the complex were identical. TenpN is a well-folded protein composed of six α-helices (helices α1 to α6) and five β-strands (strands β1 to β5) connected by loops (Fig 6a). The N-terminal region begins with a small loop, followed by a β-strand, and then a short α-helix, closely resembling the structural arrangement observed in the ToxN protein [16,17]. A long loop links helix α1 to the core β-sheet at the centre of the protein. The core of TenpN is formed by four antiparallel β strands, with β2-β3 and β4-β5 creating β hairpin motifs (Fig 6a). A short helix (α2) connects the core to the outer helix α4 through another long loop. Helix α4 is the longest helix in the TenpN structure, featuring a kink about two-thirds of the way along its length (Fig 6a). A similar analogous kinked helix was observed in the ToxN structures [16,17] and was found to provide addition interactions with antitoxin RNA holding it in place in the RNP complex.

**Fig 6:**
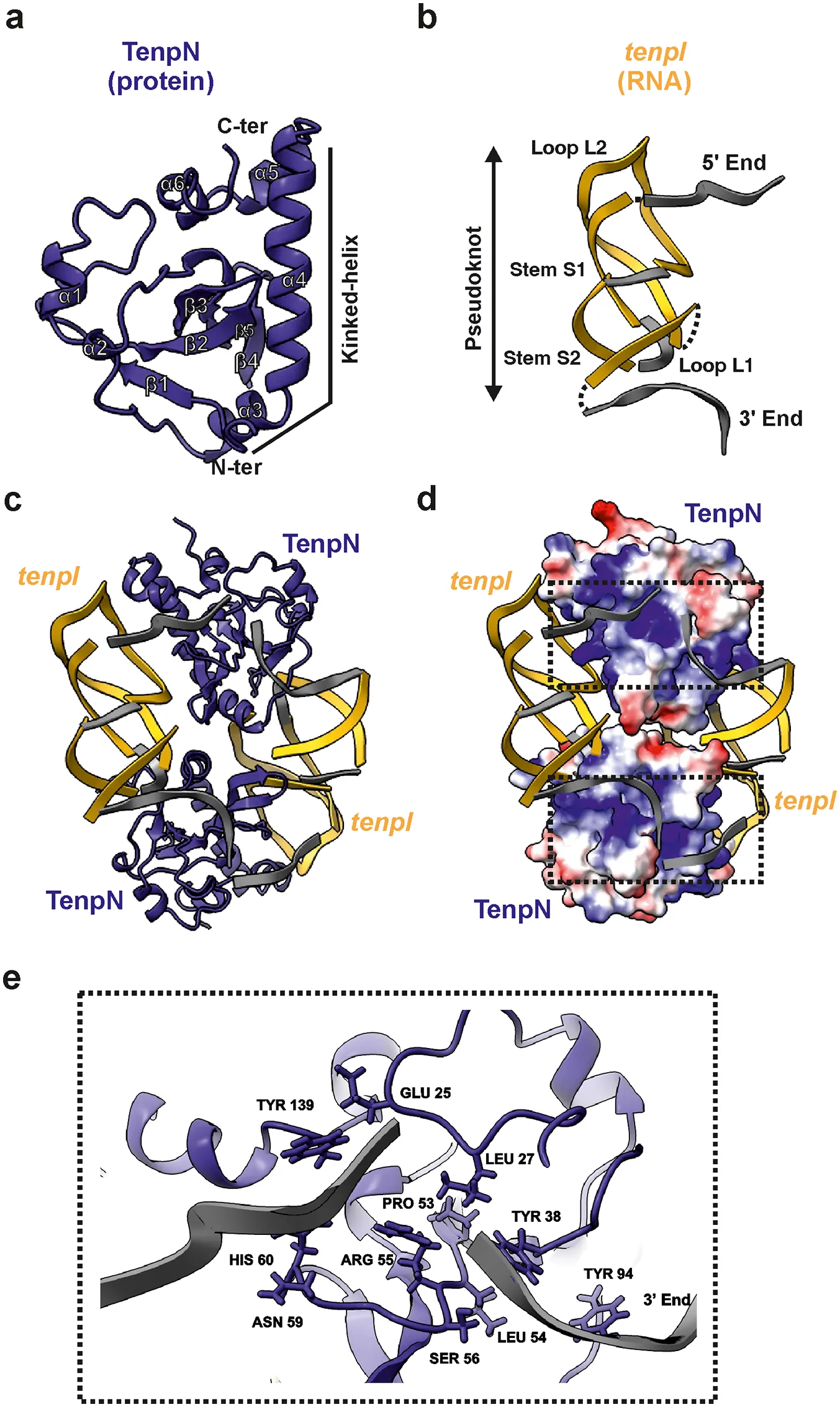
Cryo-EM structures of TenpN toxin and *tenpI* antitoxin. **a)** Structure of TenpN toxin. Toxin structure consists of a β-sheet core flanked by α-helices. Kinked-helix (α4) is marked. **b)** Structure of *tenpI* antitoxin RNA. The fitted and refined parts are shown in yellow. Parts in grey are rigid body fitted in the refined density. Dots represent the nucleotides which could not be fitted in the density. The central pseudoknot, stem S1, stem S2, loop L1, loop L2, 5’ and 3’ ends are marked. **c)** Structure of TenpIN complex. TenpN toxin and *tenpI* antitoxin are shown in blue and yellow/grey respectively. Mainly, the 5’ and 3’ single-stranded ends of *tenpI* repeats RNA bind to TenpN toxin, generating cyclic, hetero-tetrameric TenpIN assembly. **d)** Electrostatic potential surface of TenpN reveals a electropositive groove that binds the 5’ and 3’ ends of *tenpI* repeats. **e)** Snapshots of TenpIN complex showing amino acids residues which are potentially involved in processing and binding *tenpI* antitoxin repeats.

The sugar-phosphate backbone of the most part of the Rp1 RNA was traceable in the final sharpened cryo-EM maps (Fig 5d and 5e). By adjusting the contour levels, the A form helices of the central pseudoknot region could be deciphered (Fig 5e and 5f, and S9 Fig). Due to the low resolution of the structure, some parts of the RNA could not be refined in the final model, however the cryo-EM density clearly indicated the folded pseudoknot at the core of RNA, flanked by single-stranded regions at both the 5’ and 3’ ends (depicted in Fig 5e and 5f).

The *tenpI* repeat contains a central H-type pseudoknot formed with two stems (S1 and S2) separated by two loops (L1 and L2) (Fig 6b). The 5’ single stranded ends of one repeat and 3’ single stranded end of the second repeat of *tenpI* repeat bind TenpN in a head to tail manner generating a cyclic, heterotetrameric assembly (Fig 6c). The density for the single stranded 5’ and 3’ ends run along the electropositive groove on the TenpN, suggesting plausible RNA binding site and also the putative active site of the TenpN endoribonuclease (Fig 6d and 6e). The stem S1 of *tenpI* RNA is longer than the second stem S2 (Fig 2a and Fig 6b). A single nucleotide loop L1 twists and connects the two stems, co-axially stacking them one below the other (Fig 2a and 6b). A second long loop L2 runs behind the stem S1, resembling a ‘third strand’ and is longer than what is seen in the *cptI* and *toxI* RNA structures [15-17]. This loop contains several adenosines that could potentially form A-minor interactions with the first stem S1, although the exact detail of these interactions is not clear from the cryo-EM map at this resolution. In the cryo-EM density of the RNA, in loop L2, 3 adenosines at the top of the loop appear to flip outward, while the adenosines following them (lower part of L2) are pointed inward and could be involved in A-minor interactions with S1 and seen in the CptIN complex [15].

### Validation of TenpN active site by site-directed mutagenesis

We aligned the sequences of *E. coli* ToxN, CptN, and TenpN endoribonucleases, which revealed the identical and highly conserved residue positions in all three toxins (Fig 7a). These residues are likely be crucial for the structure or function of of the toxins. For example, residue positions corresponding to Gly47, Pro53, Leu54, and Ser56 in TenpN are conserved in all three toxins. Notably, TenpN Ser56 equivalent Ser53 in ToxN [16,17] and Ser52 in CptN [15] has been experimentally validated as part of the active site triad. Also, at semi-conserved conserved residue position Arg55 in TenpN equivalent to Thr52 in ToxN was also shown to be important for the activity of ToxN in *E. coli* [17], suggesting that these residues may serve a similar function in TenpN*.* Therefore, we created single-point alanine mutants of TenpN residues Pro53, Leu54, Arg55, and Ser56 (namely P53A, L54A, R55A, and S56Arespectively) in pBAD vector to check their effect on toxin’s activity monitored using bacterial growth assays.

**Fig 7:**
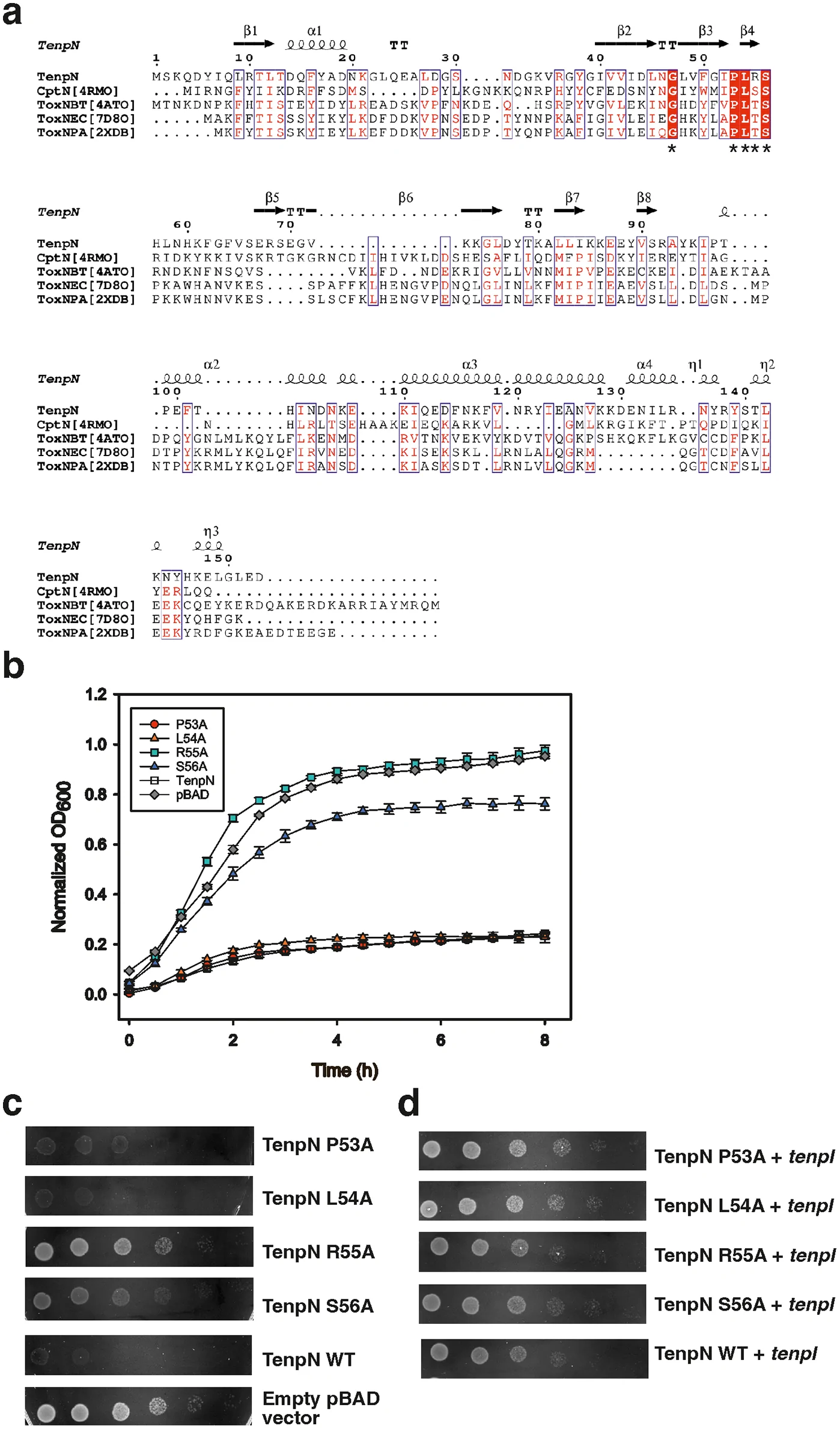
Effect of TenpN mutants on bacterial growth. **a)** Multiple sequence alignment of TenpN, ToxN, and CptN toxins whose structures have been solved. Region consisting of β2, β3, and β4 strands that constitutes the catalytic centre has several conserved residues. TenpN residues P53, L54, R55, and S56 were selected for the site-directed mutagenesis. **a)** Liquid growth assays of TenpN mutants showing that P53A and L54A mutants retain toxicity, with growth profiles similar to WT TenpN, whereas R55A and S56A mutants show reduced toxicity and growth comparable to the empty pBAD vector control. **b)** Spot growth assays of TenpN mutants corroborating the results obtained in the liquid media. Only R55A and S56A mutants exhibited rescue of growth inhibition by TenpN toxin. **c)** Antitoxin-mediated rescue of growth inhibition caused by TenpN WT and its mutants. Expression of *tenpI* in trans rescued the toxicity of WT TenpN and all mutants, resulting in growth comparable to the empty vector control.

In the liquid LB growth medium, we observed that mutants had effect on the *E. coli* growth to different extent (Fig 7b). P53A and L54A mutations in TenpN lead to the growth inhibition comparable to the WT TenpN, suggesting that these mutations do not lead to the inactivation of the toxin. However, in case of R55A and S56A we observed rescue of growth suggesting that these residues are important for TenpN activity *in vivo*. We also repeated these experiments on solid LB agar medium by performing bacterial growth spot assays (Fig 7c and 7d). In the spot assays, expression of P53A and L54A mutants pf TenpN lead to bacterial growth inhibition similar to the WT TenpN, which was rescued by the co-expression of antitoxin *toxI RNA* (Fig 7c and 7d). However, as observed in the liquid media, expression of R55A and S56A mutants lead to significant inhibition of bacterial growth (Fig 7c).

Therefore, taken together, these results show that residues R55 and S56 play roles in the activity of TenpN in bacteria. Considering their proximity to the potential cleavage site and comparison with the ToxN and CptN structures, we postulate that these residues may constitute the putative active site residues in TenpN.

## Discussion

In this study, a putative *tenpIN* TA system common to several pathogens from Enterobacteriaceae family such as *Salmonella enterica, Klebsiella pneumoniae, Shigella flexineri,* and *Escherichia coli* was functionally and structurally characterized as a type III TA system. The TenpN toxin when overexpressed in the absence of its cognate antitoxin resulted in bacterial growth inhibition. This phenotype was rescued when the sequence between the operonic promoter and terminator was co-expressed with *tenpN*, which was named as antitoxin *tenpI*. The *tenpI* consisted of two non-identical repeats (Rp1 and Rp2). Interestingly, both Rp1 and Rp2 were capable of rescuing bacterial growth inhibition caused by the toxin. Furthermore, the purified TenpIN complex from *E. coli* contained both Rp1 and Rp2 RNA molecules. Our data suggest that both Rp1 and Rp2 fold into stable structures and assemble with toxin TenpN *in vitro* into the TA RNP complex assembly.

The structure of TenpIN complex was elucidated using cryo-EM. Despite the small size of the complex (~72 kDa), the structure was resolved in its native conformation at ~ 3.9 Å resolution. The TenpIN complex is a hetero-tetramer composed of alternating two proteins and two RNA molecules in a circular, closed arrangement. Given that Rp1 provided a better fit within the density, we opted to model Rp1 for both RNA densities in the cryo-EM map. The two RNA molecules are positioned in a head-to-tail fashion, with the 5’ end of one RNA molecule close to the 3’ end of the other in the complex. The protein model was initially built using AlphaFold2 and later refined to fit into the cryo-EM density, while the RNA initial model was built *de novo*.

### *Toxin* TenpN *is similar to toxin* ToxN

Sequence alignment of ToxN, CptN, and TenpN showed that TenpN shares 13% sequence identity with both ToxN and CptN, whereas ToxN and CptN share 11% sequence identity with each other (Fig 7A). Despite significant sequence divergence, overall fold of the toxins is conserved across the three toxins.

To further explore the similarities, the available structures of CptN, ToxN, and TenpN (solved in this study) were compared using DALI [30]. DALI results showed that overall TenpN is similar to ToxN (backbone RMSD 5.4 Å) than the CptN (backbone RMSD 10.1 Å) (Fig 8a and 8b). Several common structural features were observed in TenpN and ToxN. This includes a twisted β-sheet surrounded by α-helices and a kinked helix α6 (Fig 8a). Supplementary Figure 10 shows the comparison of active site residues of ToxN and CptN with the TenpN based on the structure-based sequence alignment (S10a-S10c Fig). The active site residues in ToxN that were experimentally validated consisted of Thr 52, Ser 53 and Lys 55 (S10b Fig) while in the case of CptN, Arg 53, Ser 51 and Ser 52 (S10c Fig) formed an active site triad [15,17]. Comparing equivalent residues in TenpN structure, Arg 55, Ser 56 and His 57 may perform a similar function (S10a Fig). Indeed, using site-directed mutagenesis and bacterial growth assays, we showed that alanine mutations of Arg 55 and Ser 56, resulted in loss of TenpN activity resulting in the rescue of toxin mediated growth inhibition in bacteria (Fig 7b and 7c). Overall, these features suggest that all three toxins may function similarly as specific endoribonucleases.

**Fig 8:**
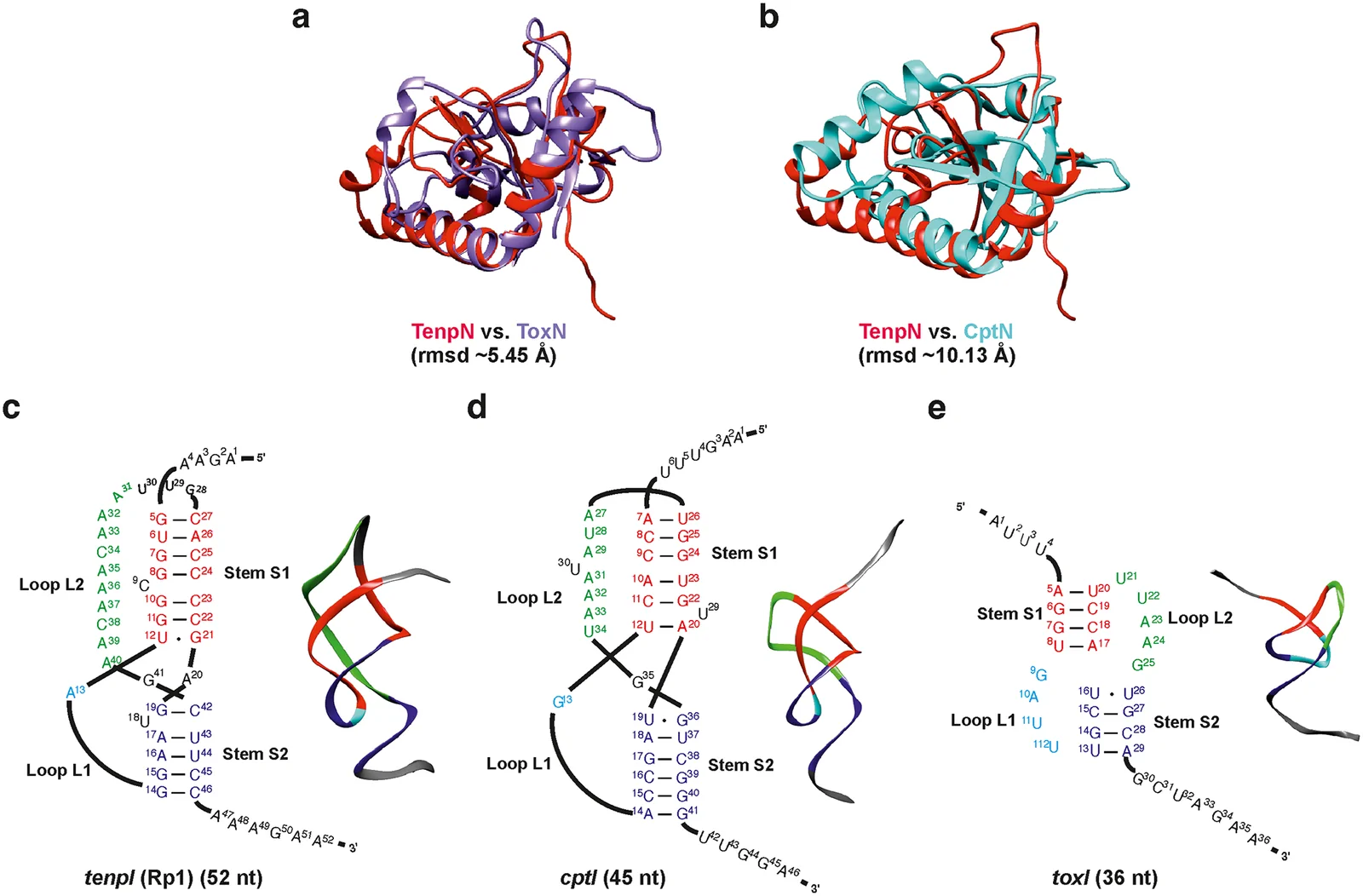
Comparison of toxin and antitoxin from TenpIN, ToxIN, and CptIN type III TA systems. **a)** Overlay of TenpN (red) and ToxN (violet, based on PDB 7D8O) structures. **b)** Overlay of TenpN (red) and CptN (cyan, based on PDB 4RMO) structures. The backbone rmsd of the two structures is mentioned in each penal. **c)** Predicted secondary structure and cryo-EM based model of *tenpI* RNA structure reported in this study. **d)** Secondary structure and structure of *cptI* antitoxin (based on PDB 4RMO). **e)** Secondary structure and structure of *toxI* antitoxin (based on PDB 7D8O).

### Antitoxin tenpI is similar to antitoxin cptI

The *tenpI* RNA repeats are usually longer than *cptI* and *toxI* RNA repeats [6]. From a structural perspective, *cptI* and *tenpI* RNAs are similar in their tertiary structures, both having two stems intertwined with two loops (Fig 8c and 8d). The single nucleotide loop L1, twists to help the second stem stack beneath the first. The loop L2 is much longer in both *cptI* and *tenpI* RNAs as compared to *toxI* RNA, running close to the first stem like a triplex, with A-rich sequences forming A-minor interactions with stem S1 base pairs. In contrast, *toxI* RNA has shorter stems and more balanced loops, each containing 4–5 nucleotides (Fig 8e). The *toxI* RNA forms a UUU triplet at the junction between the two helices. Because of the larger size, *cptI* and *tenpI* RNAs form hetero-tetrameric structures with their cognate toxins, while *toxI* forms a hetero-hexameric structure. In all the three antitoxins, the 5’ ss region of one RNA and the 3’ ss region of the other RNA converge at the active site. This forces *cptI* and *tenpI* RNAs to end their single-stranded regions in the same direction whereas the *toxI* arranges its 3’ and 5’ ends on opposite sides to accommodate three toxins and three antitoxins due to its shorter length (Fig 8c-8e).

In summary, the cryo-EM structure of TenpIN expands our understanding of type III TA systems, showing how TenpN resembles ToxN, while the *tenpI* RNA is closer to *cptI* RNA. These insights may pave the way for future studies that could use this structural knowledge to design small molecules or peptides that disrupt the complex, freeing the toxin *in vivo*, which could serve as a novel antibacterial strategy.

## Materials and methods

### Cloning and Site-directed mutagenesis

A *tenpIN* system common to *E. coli (strain ECSC033 and strain MB22* plasmid pYLMB22), *Klebsiella pneumoniae (strain* 2018C01-046, plasmid p2018C-046-5, and *strain* 2018C01-046 plasmid p2018C01-046-5), *Salmonella enterica subsp. enterica serovar (Typhimurium strain* S29 plasmid, *Kentucky strain CVM N18S0935*plasmid pN18S0935-2) was selected for cloning. Briefly, the *tenpI* was cloned with natural promoter in pRSFDuet-1 between Nco1 and BamH1 restriction sites and *tenpN* was cloned under T7 promoter between Nde1 and Xho1 restriction sites in pET-21a(+). For toxicity and spot assays, *tenpN* was cloned in pBAD vector under the inducible arabinose promoter. All the aforementioned constructs were synthesised by Genscript.

Putative antitoxin repeats 1 and 2 (Rp1 and Rp2) were created by site-directed mutagenesis from the full-length *tenpI* sequence cloned in pRSF-Duet-1 vector using the Fwd_tenpI_Rp1, Rev_tenpI_Rp1, Fwd_tenpI_Rp2, and Rev_tenpI_Rp2 primers under the natural operonic promoter (S1 Table).

The single-point alanine mutants of TenpN, namely, P53A, L54A, R55A, and S56A were generated using site-directed mutagenesis of the TenpN cloned in pBAD vector. The primers, used for creating these mutants are listed in the S1 Table. All mutants were confirmed by Sanger DNA sequencing.

### *E. coli* growth spot assays

For the spot assays, *E. coli MG1655* cells transformed either with empty pBAD/HisA vector or pBAD/HisA vector with *tenpN* alone or co-transformed with the antitoxin *tenpI* in pRSF-Deut-1 vector, were plated on Luria Bertani (LB) agar with 0.1 mg/mL ampicillin, 0.05 mg/mL kanamycin (where pRSF-Duet-1 vector was used) and 0.4% D-glucose. Primary cultures were grown from single colonies for 8–9 hours to reach growth saturation in LB media with ampicillin and/or kanamycin and 0.4% D-glucose (to repress the pBAD promoter) at 37 °C. Cells were pelleted and washed with LB containing ampicillin and/or kanamycin. After adjusting the OD_600 nm_ to ~1, cultures were serially diluted and 5 µL of each dilution was spotted on LB agar plates with ampicillin and/or kanamycin and 0.4% D-arabinose to overexpress TenpN. The plates were incubated at 37°C overnight and the results were recorded by imaging the plates.

Similar protocol was followed for the TenpN alanine mutants and controls (TenpN alone and vector only controls) to check their effect on *E. coli MG1655* growth, both in the liquid LB media as well as in the solid LB agar media as spot assays. The TenpN mutants induced growth rescue was performed by co-transforming the *tenpI* plasmid along with the TenpN mutant plasmids *in E. coli MG1655* and performing the spot assay.

### CFU experiments

To quantitate viable bacterial cells after overexpression of TenpN with either Rp1, Rp2, full length *tenpI,* cells were transformed, grown and treated like spot assay experiments. 100 µL of serially diluted cells were spread on LB agar plates containing appropriate antibiotics and incubated overnight at 37°C. The colonies were manually counted the following day to calculate CFU/mL.

### Toxicity and growth rescue assays

For liquid growth toxicity assays, transformations were performed similar to spot assays. Secondary cultures were inoculated from saturated primary cultures under toxin supressed conditions until early exponential phase. The cells were pelleted and washed and resuspended with LB media with ampicillin to adjust the OD ~ 0.2 OD_600 nm_. The cells were induced with 0.4% D-arabinose and OD_600 nm_ were measured every 1 hour for a span of 5 hours.

For toxicity rescue experiments, *tenpI* full length, Rp1 or Rp2 cloned in pRSF-Deut-1 vector were co-transformed with TenpN in pBAD/HisA vector in *E. coli MG1655* cells with ampicillin and kanamycin. Primary cultures were grown from isolated colonies. Secondary cultures were allowed to grow for about 2-2.5 hours until they reached early exponential phase. Cells were washed with LB media with appropriate antibiotics, followed by induction with L-arabinose. Cultures were induced for 2.5 hours and then washed again with LB media with antibiotics. OD_600 nm_ were adjusted to ~1 and 100 µL of serially diluted cells were spread on LB agar plates containing appropriate antibiotics and incubated overnight at 37°C. The colonies were manually counted the following day to calculate viable bacteria.

0.4% D-glucose, 0.4% L-arabinose, 0.1 mg/mL ampicillin and 0.05 mg/mL kanamycin were used throughout this study unless otherwise mentioned.

### TenpIN TA RNP complex purification

The expression and purification of the TenpIN_Ec_ TA complex was performed similar to the protocol previously described for ToxIN RNP purification [24]. Briefly, constructs containing *tenpN* with hexa-histidine tag under inducible promoter and *tenpI* under natural operonic promoter were co-transformed and co-expressed in *E. coli BL21(DE3)* Star cells. Primary culture was grown at 37°C with overnight shaking at 180 rpm. Secondary cultures were grown until OD_600 nm_ ~ 0.8 before induction of *tenpN* with 1 mM IPTG. Antitoxin was under a constitutive promoter and did not require induction. The induced culture was incubated at 37°C for 4-4.5 hours before cells were pelleted and stored at -20°C. The cells were resuspended and lysed in lysis buffer (50 mM Tris, 300 mM NaCl, 10 mM imidazole, 10% glycerol, 2 mM 2-mercaptoethanol, pH 7.5 at 25°C). The cell lysis was performed by sonication. The lysate was centrifuged at 13,000 rpm for 1 h, and the supernatant was loaded on a recharged and equilibrated Ni^2+^-NTA affinity column. The elution buffer (50 mM Tris, 300 mM NaCl, 200 mM imidazole, 10% glycerol, 2 mM 2-mercaptoethanol, pH 7.5 at 25°C) was used to elute the TenpIN TA complex. This was followed by 16–20 h of dialysis (against buffer containing 50 mM NaCl, 50 mM Tris-HCl, 1mM DTT, pH 7.5), followed by ion-exchange chromatography. TenpIN complex was eluted by increasing concentration of NaCl from 50 mM to 1 M over a volume of 100 ml. Elution peaks corresponding to TenpIN complex were pooled and further purified over size-exclusion chromatography using an S200 column (GE), equilibrated with SEC buffer (50 mM NaCl, 50 mM Tris-HCl, 1 mM DTT, pH 7.5).

### Antitoxin sequence determination from TenpIN TA complex

Fractions containing purified TenpIN TA complex after SEC were treated with proteinase K (Thermo Scientific), as per manufacturer’s instructions, to cleave TenpN protein from the complex. To extract RNA from the treated sample, chilled isopropanol, four times the volume of the sample, was added and stored at -80°C, over-night for RNA precipitation. It was subsequently centrifuged at 4 °C at 13,000 rpm for one hour. The pellets were washed with 70% chilled ethanol, followed by centrifugation at 4°C at 13,000 rpm for one hour. The air-dried pellet was resuspended in nuclease free water.

In order to increase the length of the RNA for effective Sanger sequencing, stem-loop (SL) primers [31] were used during the cDNA synthesis (SLP_Rp1 and SLP_Rp2 primers in S1 Table). First strand cDNA synthesis was performed using SuperScript III Reverse Transcriptase (Thermo Scientific) as per the manufacturer’s instructions. Briefly, first strand buffer, 0.1 M DTT and Superscript III RT were incubated with RNA obtained from the TenpIN complex at 25 °C for 5 min. This was followed by incubation at 55 °C for 60 min. The enzyme was heat-inactivated at 70 °C for 15 min. The obtained cDNA was used as the template for PCR. PCR was performed with two different sets of primers to detect both Rp1 and Rp2, namely RT_Rp1_FP, RT_Rp2_FP, and RT_Rp1 + 2_RP that had extra sequence to increase the length of cDNA (S1 Table). The primer, URPwithBamHI (universal reverse primer with BamHI site) was used as a common reverse primer corresponding to the common SL sequence that was added in the cDNA. The amplified DNA from the PCR was purified using QIAquick PCR purification kit (QIAGEN) and the antitoxin sequence was confirmed through Sanger sequencing.

### Thermal stability measurement of Rp1 and Rp2 using CD spectroscopy

To assess the thermal stability of the Rp1 and Rp2 *toxI* RNA repeats, circular dichroism (CD) spectroscopy was performed using a JASCO J-815 CD spectropolarimeter equipped with a Peltier temperature-control unit. CD spectra were recorded from 200 to 300 nm at temperatures ranging from 20 to 90°C, with measurements taken at 10°C intervals. Thermal denaturation profiles were subsequently obtained by monitoring the ellipticity at 265 nm while increasing the temperature from 10 to 90°C in 1°C increments at a heating rate of 1°C/min. The resulting temperature-dependent ellipticity data were normalized and fitted to a sigmoid function to determine the melting temperature (Tm) of each RNA repeat.

### Negative staining TEM sample preparation and imaging

To evaluate the homogeneity and particle distribution of the TenpIN TA RNP complex, negative staining transmission electron microscopy (NS-TEM) was used. The formvar carbon-coated copper grids (EM grid, 300 mesh; TedPella) were glow discharged for 30 s at a negative polarity using a GlowQube system (Quorum Technologies Ltd.) to enhance hydrophilicity of the carbon support film. The different peak fractions were diluted 40x times with the SEC buffer (See TenpIN purification in Materials and Methods section) to visualize under room temperature TEM. Four microliters of various diluted SEC fractions (0.1 mg/mL) were placed on the glow-discharged grids and blotted with Whattman filter paper after 60 s incubation. Samples containing the grids were stained with 1% uranyl acetate (Polysciences Inc. Warrington, PA, USA) followed by blotting and air drying for 2 minutes. The TEM data were collected at room temperature using a 120 kV FEI Tecnai T12 electron microscope, equipped with a side-mounted Olympus VELITA (2Kx2K) CCD camera, at 80,000X magnification and a calibrated pixel size of 2.54 Å/pixel. In moderately stained area on the grid, a total of 30 images were collected. The images were collected using a side-mounted Olympus VELITA (2000 x 2000) charge-coupled device camera.

### Negative staining TEM data processing of TenpIN

The micrographs were imported into EMAN2.1 [32] for further analysis. In total 6000 particles were picked automatically, and their coordinates were extracted using the e2boxer.py tool in the EMAN2.1 software. The general stack tool was used to stack the particles with EMAN and reference free 2D class averages were generated for the stacked particles using e2refine2d.py script. For further processing, a high pass filter of 0.2 was applied during class averaging and the particle stack was exported to Relion 2.1 to perform 2D class averaging. In the first round of 2D classification, the particles were split into 50 classes and the classes with good signal to noise ratio were chosen for the subsequent round to class averaging by splitting them into 30 classes. Finally, 3000 particles contributing to the best classes after converging with different particle views were selected to execute the last round of 2D class averaging using simple_prime2D of SIMPLE 2.1 software [33] with a mask diameter of 80 pixels at 2.54 Å per pixel value.

### Cryo-EM sample preparation of TenpIN

QUANTIFOIL Copper 300 mesh grids with R 1.2/1.3 size were glow discharged for 90 s at 20 mA using Quorum GlowCube. Three microliters of purified TenpIN complex were placed on the glow discharged grids and incubated for 10 s before blotting for 8 s at 100% humidity. The sample containing grids were flash frozen by quickly plunging into the liquid ethane using a FEI Vitrobot IV plunger (Thermo Fisher Scientific). The grids were stored in liquid nitrogen until they were used for data collection.

### Cryo-EM data acquisition of TenpIN using 200 kV Talos Arctica

Talos Arctica 200kV cryo-TEM (Thermo Fisher Scientific) was used for cryo-EM data acquisition of TenpIN TA complex. The instrument was equipped with K2 direct electron detector (Gatan Inc.). A total of 2855 movie files were collected using the Latitude-S automated data acquisition tool (Gatan Inc.). Each movie file contained 20 frames with a total electron dose of 50 e-/Å² with a defocus range of -0.75 to -2.25 µm. The data were collected at 54,000X magnification with the calibrated pixel size of 0.92 Å (S2 Table).

### Cryo-EM data processing of TenpIN

Micrographs were manually screened in cisTEM software and those with poor signal-to-noise ratio were discarded. Data was processed using RELION 3.1.2 software while beam-induced motion correction was carried out on the movie files using the MotionCor2 package. The contrast transfer function (CTF) estimation of the micrographs was performed using CTFFIND 4.1.13. For the generation of a 2D class average template for automated particle picking, initially around 10000 particles were manually picked and reference-free 2D classification was performed using RELION 3.1.2 to choose the best 2D class averages. In RELION, 1,146,611 particles were auto-picked using the reference 2D classes. Automatically picked particles were extracted with a box size of 220 pixels. Multiple rounds of 2D classification were run with the extracted particles to clean the particle stack. Around 8500 particles contributing to different orientations of TenpIN were selected to generate an *ab initio* initial model. The best 5,42,913 particles from the dataset were 3D classified into 8 classes using *ab initio* map as reference. The 3D classification was performed with C1 and C2 symmetry separately. In asymmetric 3D classification, a 3D class with 258549 particles was observed as a well-resolved map. 3D auto refinement was carried out for the 3D map using a soft mask in RELION 3.1.2. Subsequently, per-particle defocus refinement with beam tilt correction, anisotropic magnification correction, and per micrograph astigmatism fitting were performed. Finally, the corrected particles were polished in RELION using Bayesian polish. The polished particles were subjected to final 3D refinement and sharpening. The 3D classification with C2 symmetry generated a class with a well-resolved map contributed by 327240 particles. Refinements and post-processing are carried out similarly to C1 symmetry map. The final map was sharpened with Phenix which was provided as the input for DeepEMhancer [34] to obtain the final density map.

The structural differences between symmetric and asymmetric reconstruction were analyzed after superimposing both the cryo-EM maps in UCSF Chimera and UCSF ChimeraX.

### Model building and fitting of TenpIN

The high confidence model of TenpN generated using AlphaFold2 was used as initial coordinates and the final model was refined using Phenix and Coot software iteratively.

The *tenpI* RNA model was built *de novo*. The secondary structure for both Rp1 and Rp2 were built manually taking cues from various RNA secondary structure prediction tools such as SSRTool, RNAComposer, and FARFAR2 [34–36]. Furthermore, the RNA structure was built through fragment-based approach using Chimera and Coot. Additionally, models generated from simRNA web2.0 [29], and RhoFold+ [37] were used to fit into the density map. Fragments generated *de novo* and the predicted models were corrected and modelled wherever necessary and used for flex-EM fitting using ccp4-EM and refinement using Coot (S2 Table). The quality of the final model was assessed using Coot [38], the Molprobity server [39], and the validation server [40].

### Mapping of conserved residues on type III toxins

Representative toxin structure from each family, ToxN, CptN, and TenpN (this study) were considered for sequence and structure-based analysis. Sequence alignments were performed using MAFFT [41]. The alignment was generated using ESPript 3.2 [42]. For structural alignment, DALI (Protein structure comparison server) was used [30]. Equivalent residues were deduced using structure guided alignment and visualization using Pymol [43].

## Supporting information

S1 Fig BLAST analysis of the *E. coli* TenpN sequence. BLASTN analysis showing 100% nucleotide sequence identity between the *E. coli* TenpN sequence used as the query and putative TenpN sequences identified in several pathogenic bacteria, including *Klebsiella pneumoniae*, *Salmonella enterica*, *Shigella flexneri*, and multiple *Escherichia coli* strains.
(TIF)

S2 Fig Purification and characterization of TenpIN complex. a) 12% SDS-PAGE stained with Coomassie blue dye, showing presence of TenpN toxin protein after His-tag affinity column purification. b) 15% Urea-PAGE stained with toluidine blue dye, showing presence of *tenpI* antitoxin RNA after His-tag affinity column purification. c) Anion-exchange chromatography (using MonoQ column) profile of TenpIN purification. Small quantity of free TenpN elutes in the flowthrough under low salt condition. TenpIN complex elutes at around 500 mM NaCl concentration. d) TenpIN complex eluted from ion-exchange column was further purified over a size-exclusion column (S200 Superdex column). TenpIN complex elutes as tetramer (approximate molecular weight size of ~72 kDa). e) Free TenpN complex eluted from ion-exchange column was further purified over a size-exclusion column (S200 Superdex column). Free TenpN elutes as monomer (approximate molecular weight size of ~18 kDa). f) 15% Urea-PAGE stained with toluidine blue dye showing *in vitro* transcribed and purified Rp1 and Rp2 RNAs.
(TIF)

S3 Fig Characterization of *in vitro* made Rp1 and Rp2 *tenpI* RNA repeats using CD spectroscopy. a) CD spectra of Rp1 at different temperatures ranging from 20 to 90 °C. b) CD spectra of Rp2 at different temperatures ranging from 20 to 90 °C. c) Thermal melting profile of Rp1 and Rp1 measured by monitoring CD signal at 265 nm from 10 to 90 °C. The obtained melting temperatures (Tm) of Rp1 and Rp2 are mentioned in the figure.
(TIF)

S4 Fig Analytical size-exclusion chromatography (SEC) analysis of *in vitro* assembled TenpN – Rp1 and TenpN – Rp2 complexes. Overlay of SEC elution profiles of free TenpN (black), TenpN – Rp1 complex (red), and TenpN – Rp2 complex (blue).
(TIF)

S5 Fig Negative-stained TEM micrograph of SEC fractions. Micrograph from the left slope of the elution peak of purified TenpIN complex showing homogenous particles of the sample. Well-defined class averages indicate that TenpIN sample has a stable and ordered structure.
(TIF)

S6 Fig Negative-stained TEM micrographs of SEC fractions. Micrograph from the middle (a) and right slope (b) of the elution peak of purified TenpIN complex showing heterogeneity of the sample.
(TIF)

S7 Fig Cryo-EM data processing workflow of TenpIN with C2 symmetry. The angular distribution, local resolution of the map, and the FSC curve are shown in the image.
(TIF)

S8 Fig Fitting of Rp1 (a) vs. Rp2 (b) models in the cryo-EM density. Only the fitting of the stems and a loop regions is only shown for clarity. Rp1 model showed better fitting than Rp2 model, which is also reflected in the Correlation coefficient (CC) volume number.
(TIF)

S9 Fig Rp1 *tenpI* antitoxin model fitted into the refined cryo-EM density. The helical regions (stem 1 and stem 2) and loop 2 of Rp1 can be easily visualized in the refined density.
(TIF)

S10 Fig Conserved and active site residues in TenpN (a), ToxN (b), and CptN (c) toxins. (TIF)

S1 Table List of primers used in this study. (DOCX)

S2 Table Cryo-EM Data collection, image processing and refinement for TenpIN. (DOCX)

S1 File Graphical abstract. (DOCX)

## Abbreviations

TA: toxin-antitoxin
RNP complex: RNA-protein complex
*toxIN*: toxin and inhibitor of toxin
*cptIN*: CoPrococcus Type III Inhibitor/toxiN
*tenpIN*: Type III ENdogenous to Photorhabdus Inhibitor/toxiN

## References

[1] Page R, Peti W. Toxin-antitoxin systems in bacterial growth arrest and persistence. Nat Chem Biol. 2016;12(4):208–14. doi: 10.1038/nchembio.2044 26991085

[2] Pizzolato-Cezar LR, Spira B, Machini MT. Bacterial toxin-antitoxin systems: Novel insights on toxin activation across populations and experimental shortcomings. Curr Res Microb Sci. 2023;5:100204. doi: 10.1016/j.crmicr.2023.100204 38024808 PMC10643148

[3] Harms A, Brodersen DE, Mitarai N, Gerdes K. Toxins, targets, and triggers: an overview of toxin-antitoxin biology. Molecular Cell. 2018;70(5):768–84. doi: 10.1016/j.molcel.2018.01.003 29398446

[4] Jurėnas D, Fraikin N, Goormaghtigh F, Van Melderen L. Biology and evolution of bacterial toxin-antitoxin systems. Nat Rev Microbiol. 2022;20(6):335–50. doi: 10.1038/s41579-021-00661-1 34975154

[5] LeRoux M, Laub MT. Toxin-Antitoxin Systems as Phage Defense Elements. Annu Rev Microbiol. 2022;76:21–43. doi: 10.1146/annurev-micro-020722-013730 35395167

[6] Blower TR, Short FL, Rao F, Mizuguchi K, Pei XY, Fineran PC, et al. Identification and classification of bacterial Type III toxin-antitoxin systems encoded in chromosomal and plasmid genomes. Nucleic Acids Res. 2012;40(13):6158–73. doi: 10.1093/nar/gks231 22434880 PMC3401426

[7] Dutta H, Noor S, Nadig K, Singh M. Structural and Functional Diversity of RNA-Containing Toxin-Antitoxin Systems. Wiley Interdiscip Rev RNA. 2026;17(3):e70042. doi: 10.1002/wrna.70042 42077024

[8] Blower TR, Chai R, Przybilski R, Chindhy S, Fang X, Kidman SE, et al. Evolution of Pectobacterium Bacteriophage ΦM1 To Escape Two Bifunctional Type III Toxin-Antitoxin and Abortive Infection Systems through Mutations in a Single Viral Gene. Appl Environ Microbiol. 2017;83(8):e03229-16. doi: 10.1128/AEM.03229-16 28159786 PMC5377504

[9] Chen B, Akusobi C, Fang X, Salmond GPC. Environmental T4-Family Bacteriophages Evolve to Escape Abortive Infection via Multiple Routes in a Bacterial Host Employing “Altruistic Suicide” through Type III Toxin-Antitoxin Systems. Front Microbiol. 2017;8:1006. doi: 10.3389/fmicb.2017.01006 28620370 PMC5449768

[10] Schuster CF, Bertram R. Toxin-Antitoxin Systems of Staphylococcus aureus. Toxins (Basel). 2016;8(5):140. doi: 10.3390/toxins8050140 27164142 PMC4885055

[11] Nadig K, Sandhya S, Singh M. Bioinformatic exploration reveals features of tenpIN family of type III toxin-antitoxin systems in bacteria and viruses. Sci Rep. 2025;15(1):26624. doi: 10.1038/s41598-025-04853-0 40695854 PMC12283969

[12] Daruka L, Czikkely MS, Szili P, Farkas Z, Balogh D, Grézal G, et al. ESKAPE pathogens rapidly develop resistance against antibiotics in development in vitro. Nat Microbiol. 2025;10(2):313–31. doi: 10.1038/s41564-024-01891-8 39805953 PMC11790497

[13] Srivastava A, Pati S, Kaushik H, Singh S, Garg LC. Toxin-antitoxin systems and their medical applications: current status and future perspective. Appl Microbiol Biotechnol. 2021;105(5):1803–21. doi: 10.1007/s00253-021-11134-z 33582835

[14] Lin J, Guo Y, Yao J, Tang K, Wang X. Applications of toxin-antitoxin systems in synthetic biology. Eng Microbiol. 2023;3(2):100069. doi: 10.1016/j.engmic.2023.100069 39629251 PMC11610964

[15] Rao F, Short FL, Voss JE, Blower TR, Orme AL, Whittaker TE, et al. Co-evolution of quaternary organization and novel RNA tertiary interactions revealed in the crystal structure of a bacterial protein-RNA toxin-antitoxin system. Nucleic Acids Res. 2015;43(19):9529–40. doi: 10.1093/nar/gkv868 26350213 PMC4627078

[16] Blower TR, Pei XY, Short FL, Fineran PC, Humphreys DP, Luisi BF, et al. A processed noncoding RNA regulates an altruistic bacterial antiviral system. Nat Struct Mol Biol. 2011;18(2):185–90. doi: 10.1038/nsmb.1981 21240270 PMC4612426

[17] Manikandan P, Sandhya S, Nadig K, Paul S, Srinivasan N, Rothweiler U, et al. Identification, functional characterization, assembly and structure of ToxIN type III toxin-antitoxin complex from E. coli. Nucleic Acids Res. 2022;50(3):1687–700. doi: 10.1093/nar/gkab1264 35018473 PMC8860590

[18] Short FL, Pei XY, Blower TR, Ong S-L, Fineran PC, Luisi BF, et al. Selectivity and self-assembly in the control of a bacterial toxin by an antitoxic noncoding RNA pseudoknot. Proc Natl Acad Sci U S A. 2013;110(3):E241-9. doi: 10.1073/pnas.1216039110 23267117 PMC3549085

[19] Samson JE, Spinelli S, Cambillau C, Moineau S. Structure and activity of AbiQ, a lactococcal endoribonuclease belonging to the type III toxin-antitoxin system. Mol Microbiol. 2013;87(4):756–68. doi: 10.1111/mmi.12129 23279123

[20] Hsueh BY, Severin GB, Elg CA, Waldron EJ, Kant A, Wessel AJ, et al. Phage defence by deaminase-mediated depletion of deoxynucleotides in bacteria. Nat Microbiol. 2022;7(8):1210–20. doi: 10.1038/s41564-022-01162-4 35817890 PMC9830645

[21] Fineran PC, Blower TR, Foulds IJ, Humphreys DP, Lilley KS, Salmond GPC. The phage abortive infection system, ToxIN, functions as a protein-RNA toxin-antitoxin pair. Proc Natl Acad Sci U S A. 2009;106(3):894–9. doi: 10.1073/pnas.0808832106 19124776 PMC2630095

[22] Benson G. Tandem repeats finder: a program to analyze DNA sequences. Nucleic Acids Res. 1999;27(2):573–80. doi: 10.1093/nar/27.2.573 9862982 PMC148217

[23] Dutta H, Manikandan P, Singh M. Role of Antitoxin RNA Pseudoknot in Regulating Toxin Activity and Toxin-antitoxin RNP Complex Assembly. J Mol Biol. 2025;437(21):169410. doi: 10.1016/j.jmb.2025.169410 40939421

[24] Manikandan P, Nadig K, Singh M. Large-scale Purification of Type III Toxin-antitoxin Ribonucleoprotein Complex and its Components from Escherichia coli for Biophysical Studies. Bio Protoc. 2023;13(13):e4763. doi: 10.21769/BioProtoc.4763 37456336 PMC10338634

[25] Jumper J, Evans R, Pritzel A, Green T, Figurnov M, Ronneberger O, et al. Highly accurate protein structure prediction with AlphaFold. Nature. 2021;596(7873):583–9. doi: 10.1038/s41586-021-03819-2 34265844 PMC8371605

[26] Emsley P, Lohkamp B, Scott WG, Cowtan K. Features and development of Coot. Acta Crystallogr D Biol Crystallogr. 2010;66(Pt 4):486–501. doi: 10.1107/S0907444910007493 20383002 PMC2852313

[27] Pettersen EF, Goddard TD, Huang CC, Couch GS, Greenblatt DM, Meng EC, et al. UCSF Chimera--a visualization system for exploratory research and analysis. J Comput Chem. 2004;25(13):1605–12. doi: 10.1002/jcc.20084 15264254

[28] Afonine PV, Grosse-Kunstleve RW, Echols N, Headd JJ, Moriarty NW, Mustyakimov M, et al. Towards automated crystallographic structure refinement with phenix.refine. Acta Crystallogr D Biol Crystallogr. 2012;68(Pt 4):352–67. doi: 10.1107/S0907444912001308 22505256 PMC3322595

[29] Moafinejad SN, de Aquino BRH, Boniecki MJ, Pandaranadar Jeyeram IPN, Nikolaev G, Magnus M, et al. SimRNAweb v2.0: a web server for RNA folding simulations and 3D structure modeling, with optional restraints and enhanced analysis of folding trajectories. Nucleic Acids Res. 2024;52(W1):W368–73. doi: 10.1093/nar/gkae356 38738621 PMC11223799

[30] Holm L, Rosenström P. Dali server: conservation mapping in 3D. Nucleic Acids Res. 2010;38(Web Server issue):W545-9. doi: 10.1093/nar/gkq366 20457744 PMC2896194

[31] Kramer MF. Stem-loop RT-qPCR for miRNAs. Curr Protoc Mol Biol. 2011;Chapter 15:Unit 15.10. doi: 10.1002/0471142727.mb1510s95 21732315 PMC3152947

[32] Tang G, Peng L, Baldwin PR, Mann DS, Jiang W, Rees I, et al. EMAN2: an extensible image processing suite for electron microscopy. J Struct Biol. 2007;157(1):38–46. doi: 10.1016/j.jsb.2006.05.009 16859925

[33] Elmlund D, Elmlund H. SIMPLE: Software for ab initio reconstruction of heterogeneous single-particles. J Struct Biol. 2012;180(3):420–7. doi: 10.1016/j.jsb.2012.07.010 22902564

[34] Sanchez-Garcia R, Gomez-Blanco J, Cuervo A, Carazo JM, Sorzano COS, Vargas J. DeepEMhancer: a deep learning solution for cryo-EM volume post-processing. Commun Biol. 2021;4(1):874. doi: 10.1038/s42003-021-02399-1 34267316 PMC8282847

[35] Sarzynska J, Popenda M, Antczak M, Szachniuk M. RNA tertiary structure prediction using RNAComposer in CASP15. Proteins. 2023;91(12):1790–9. doi: 10.1002/prot.26578 37615316

[36] Watkins AM, Rangan R, Das R. FARFAR2: Improved De Novo Rosetta Prediction of Complex Global RNA Folds. Structure. 2020;28(8):963–76.e6. doi: 10.1016/j.str.2020.05.011 32531203 PMC7415647

[37] Shen T, Hu Z, Sun S, Liu D, Wong F, Wang J, et al. Accurate RNA 3D structure prediction using a language model-based deep learning approach. Nat Methods. 2024;21(12):2287–98. doi: 10.1038/s41592-024-02487-0 39572716 PMC11621015

[38] Emsley P, Cowtan K. Coot: model-building tools for molecular graphics. Acta Crystallogr D Biol Crystallogr. 2004;60(Pt 12 Pt 1):2126–32. doi: 10.1107/S0907444904019158 15572765

[39] Chen VB, Arendall WB 3rd, Headd JJ, Keedy DA, Immormino RM, Kapral GJ, et al. MolProbity: all-atom structure validation for macromolecular crystallography. Acta Crystallogr D Biol Crystallogr. 2010;66(Pt 1):12–21. doi: 10.1107/S0907444909042073 20057044 PMC2803126

[40] Smart OS, Horský V, Gore S, Svobodová Vařeková R, Bendová V, Kleywegt GJ, et al. Worldwide Protein Data Bank validation information: usage and trends. Acta Crystallogr D Struct Biol. 2018;74(Pt 3):237–44. doi: 10.1107/S2059798318003303 29533231 PMC5947764

[41] Rozewicki J, Li S, Amada KM, Standley DM, Katoh K. MAFFT-DASH: integrated protein sequence and structural alignment. Nucleic Acids Res. 2019;47(W1):W5–10. doi: 10.1093/nar/gkz342 31062021 PMC6602451

[42] Robert X, Gouet P. Deciphering key features in protein structures with the new ENDscript server. Nucleic Acids Res. 2014;42(Web Server issue):W320-4. doi: 10.1093/nar/gku316 24753421 PMC4086106

[43] The PyMOL Molecular Graphics System Vrp, Schrödinger, LLC. The PyMOL Molecular Graphics System, Version 1.2r3pre. Schrödinger, LLC.

