# Supplementary material for: Cryo-EM reveals the quaternary architecture of TenpIN type III toxin-antitoxin RNP complex common to pathogenic bacteria
Source: PLoS Pathog. 2026 Oct 6;22(10):e1014690. doi: 10.1371/journal.ppat.1014690 (PMC13682666; doi:10.1371/journal.ppat.1014690)
Supplement: Supplementary file 1 [file ppat.1014690.s011.docx]

| **Primer name** | **Sequence** | **Number of nucleotides** |
| --- | --- | --- |
| Fwd_tenpI_Rp1 | GAAAGATGTGCAAAGCCCTCTCTTACAGAGG | 31 |
| Rev_tenpI_Rp1 | GGGCTTTGCACATCTTTCTTTGGAAGCTTGTTTG | 34 |
| Fwd_tenpI_Rp2 | GTAAATCTCTTCCAAAGAAAGATGTGCGGTAGAG | 34 |
| Rev_tenpI_Rp2 | TTTCTTTGGAAGAGATTTACTTATATCAAAAATTATATGTG | 41 |
| SLP_Rp1 | GTCGTATCCAGTGCAGGGTCCGAGGTATTCGCACTGGATACGACTTCTTTGGTTG | 55 |
| SLP_Rp2 | GTCGTATCCAGTGCAGGGTCCGAGGTATTCGCACTGGATACGACTTCGTTGAGTC | 55 |
| RT_Rp1_FP | CATGCCATGGCATATAATTTTTGATATAAGTAAATCTAGAAGTGGCGGTAGG | 52 |
| RT_Rp2_FP | CATGCCATGGCATATAATTTTTGATATAAGTAAATCT AGATGTGCGGTAG | 50 |
| RT_Rp1+2_RP | GTACGGATCCGCCCTCTGTAAGAGAGGGCTTTGCGTCGTATCCAGTGCAG | 50 |
| URP with BamHI | GTACGGATCCCCAGTGCAGGGTCCGAGGTAT | 31 |
| Seq_FP_Rp1 | CATGCCATGGCATATAATTTTTGATATAAG | 30 |
| Seq_RP_Rp1,2 | GTACGGATCCGCCCTCTGTAAG | 22 |
| P53Afwd | GGCATTGCGCTGCGTAGCCACCTGAACC | 28 |
| P53Arev | GCAGCGCAATGCCAAAAACCAGACCGTTC | 29 |
| L54Afwd | ATTCCGGCGCGTAGCCACCTGAACCACAAG | 30 |
| L54Arev | CTACGCGCCGGAATGCCAAAAACCAGACCGTTC | 33 |
| R55Afwd | CCGCTGGCGAGCCACCTGAACCACAAGTTC | 30 |
| R55Arev | GTGGCTCGCCAGCGGAATGCCAAAAACCAGACC | 33 |
| S56Afwd | CTGCGTGCGCACCTGAACCACAAGTTCGGC | 30 |
| S56Arev | CAGGTGCGCACGCAGCGGAATGCCAAAAACC | 31 |
| H57Afwd | CGTAGCGCGCTGAACCACAAGTTCGGCTTTG | 31 |
| H57Arev | GTTCAGCACGCTACGCAGCGGAATGCCAAAAACC | 34 |
