# Supplementary material for: Cryo-EM reveals the quaternary architecture of TenpIN type III toxin-antitoxin RNP complex common to pathogenic bacteria
Source: PLoS Pathog. 2026 Oct 6;22(10):e1014690. doi: 10.1371/journal.ppat.1014690 (PMC13682666; doi:10.1371/journal.ppat.1014690)
Supplement: Supplementary file 2 [file ppat.1014690.s012.docx]

| **Data collection and processing** | **TenpIN – 200kV** |
| --- | --- |
| Magnification | 54000x |
| Voltage | 200kV |
| Electron exposure (e^-^/Å^2^) | 50 e-/Å² |
| Defocus range (µm) | -0.75 μm to -2.25 µm |
| Pixel size (Å) | 0.92 Å |
| Symmetry Imposed | C2 |
| Number of Particles | 327240 |
| Map Resolution (Å) | 3.9 Å |
| FSC threshold | 0.143 |
| Map Sharpening B Factor (Å^2^) | -75 Å |
| Number of Movies | 2855 |
| **Cryo-EM refinement statistics** | |
| **Validation** | **TenpIN** |
| MolProbity score | 2.38 |
| Clash score | 28 |
| CC mask | 0.67 |
| **Ramachandran plot** | **TenpN** |
| Favored (%) | 93.71 |
| Allowed (%) | 6.29 |
| Outliers (%) | 0.00 |
| CC mask | 0.67 |
