# Supplementary material for: Cryo-EM reveals the quaternary architecture of TenpIN type III toxin-antitoxin RNP complex common to pathogenic bacteria
Source: PLoS Pathog. 2026 Oct 6;22(10):e1014690. doi: 10.1371/journal.ppat.1014690 (PMC13682666; doi:10.1371/journal.ppat.1014690)
Supplement: Supplementary file 4 [file ppat.1014690.s015.docx]

**Point by point response to the reviewers comments:**

**Cryo-EM reveals the quaternary architecture of TenpIN type III toxin-antitoxin RNP complex common to pathogenic bacteria**

Kavyashree Nadig^1,$^, Surekha Padmanaban.^1,$^, Salik Noor^1,$^, Sankaran Sandhya^1^, Parthasarathy Manikandan^1,2^, Somnath Dutta^1,*^, and Mahavir Singh^1,*^

**Part I – Summary**

*Please use this section to discuss strengths/weaknesses of study, novelty/significance, general execution and scholarship.*

***Reviewer #1:*** *In this manuscript, the authors characterize a type III toxin–antitoxin system, TenpIN, which is reported to be universally present in the Enterobacteriaceae family. Bacterial growth and rescue assays were performed to demonstrate the toxicity of the TenpN protein and the antitoxin activity of RNA repeats. The structure of the TenpN–RNA complex was determined at ~4 Å resolution using cryo-EM, revealing a heterotetrameric arrangement. However, given the relatively low resolution, detailed protein/RNA interactions cannot be resolved, which significantly limits the overall scientific impact of the study.*

**Authors’ response:** We thank the reviewer for providing a fair assessment of our manuscript. We agree with the reviewer that due to the lower resolution of the structure, details of protein-RNA interactions are not clear. This limitation was mainly due to the small size (~72 kDa) of the complex and limited resources at our end. Therefore, we refrained from making a strong claim on the protein/RNA interactions in detail. Nonetheless, this is the first time TenpIN complex structure is solved. It revealed the quaternary assembly of the complex and also the secondary structural features of protein and the RNA are very evident. Based on the structure we have suggested putative active site residues which have been validated using site-directed mutagenesis approach. We believe that we have learned significantly about the structure and assembly of the first TenpIN complex from bacteria, which will be of interest to the readers of PLOS Pathogens and can help the future studies of this TenpIN complex.

***Reviewer #2:*** *This manuscript reports the first structural characterization of a TenpIN type III toxin–antitoxin complex and therefore addresses a genuine gap in the field. The combination of functional assays in E. coli, biochemical purification, and cryo-EM reconstruction supports the central claim that the selected TenpIN system forms a 2:2 protein:RNA assembly and that both non-identical RNA repeats are functionally competent antitoxins. The work is potentially publishable, and the structural finding is of clear interest. However, in its current form, the manuscript overstates several conclusions, leaves key mechanistic points insufficiently supported, and contains a number of textual inconsistencies, methodological ambiguities, and typographical problems that need careful revision. In my view, the manuscript contains a structurally interesting dataset and could become suitable for publication after substantial revision, but several of the current mechanistic and structural interpretations need to be either experimentally strengthened or more carefully delimited*.

**Authors’ response:** We thank the reviewer for the valuable comments and assessment of our manuscript. We have worked on all the suggestions of the reviewer and provided additional data in the revised manuscript. A point-by-point response to all the queries raised by both the reviewers is provided below.

**Part II – Major Issues: Key Experiments Required for Acceptance**

*Please use this section to detail the key new experiments or modifications of existing experiments that should be absolutely required to validate study conclusions.*

*Generally, there should be no more than 3 such required experiments or major modifications for a "Major Revision" recommendation. If more than 3 experiments are necessary to validate the study conclusions, then you are encouraged to recommend "Reject".*

***Reviewer #1:***

*1. No cryo-EM density map has been provided to the reviewers, making it difficult to assess the quality of the reconstruction and the agreement between the model and the map.*

**Authors’ response:** We had provided the EMDB and PDB validation reports during the submission of our manuscript to the submission portal. Also, the cryoEM maps and PDB models are successfully deposited to the EMDB (accession code EMD-64795) and PDB (accession code 9V7X) databases. We now provided the cryo-EM map and PDB file along with the EMDB and PDB validation reports and hope they these files will be made accessible to the reviewers.

Additionally, Figures 4 and 5 and **new Supplementary Figures S8 and S9** show the quality of the maps and the models.

*2. The authors state that the “left shoulder” of the SEC peak was used for cryo-EM analysis because it behaves markedly differently from the “central” and “right” portions of the peak. However, no details are provided regarding which elution fractions correspond to each region. Additionally, based on Supplementary Figure S3, only the TenpN/Rp2 complex appears to exhibit a left shoulder. Was this the sample used for the cryo-EM study? If so, why was Rp1 used for model building instead of Rp2? Furthermore, on page 8, the authors state that “cryo-EM sample preparation involved collecting particles from a specific fraction after SEC (from the right shoulder of the elution peak),” which is inconsistent with the earlier description and therefore confusing.*

**Authors’ response:** We thank the review for pointing this out. We have now clarified it.

For the cryo-EM, we used the samples of the TenpIN complex purified after co-expression of TenpN and *tenpI in*  *E. coli*. Please refer to SI Figure S2d. The left slope, centre, and right slope (we now refer shoulder to slope) here corresponds to the SEC fractions during purification of the TenpIN complex achieved on a preparatory grade Superdex S200 column (Cytiva). The negative-stained micrographs had revealed homogenous particles from the left shoulder (~fractions 48, elution volume ~ 73 ml) than the central (~ fraction 50, column volume ~ 75 ml) and right shoulder (fraction 56-57, elution volume ~ 77 ml) (SI Figure S2d and SI Figures S5 and S6).

The data shown in SI Figure S4, shows that *in vitro* transcribed and purified Rp1 and Rp2 can assemble into RNP complexes with the TenpN protein. This experiment was performed on an analytical SEC column and these samples were not used for the cryo-EM analysis.

We also thank the reviewer for identifying the inconsistency on page 8. We have now re-written this section.

***Reviewer #2:***

***1.*** *A central issue is that the final structural interpretation relies on fitting Rp1 into both RNA densities, even though the authors state that the purified complex contains both Rp1 and Rp2. Their rationale is indirect: Rp1 fits better, Rp2 is shorter by three nucleotides, Rp2 rescue appears somewhat weaker in spot assays, and the authors speculate that the cryo-EM fraction may be enriched for Rp1-containing complexes. This is plausible, but still not definitive. As written, the manuscript risks giving the impression that the structure represents the biologically dominant state, while the data seem more consistent with a model of one plausible repeat-derived density assignment rather than an unambiguous Rp1-only structure. The claims should therefore be softened unless the authors can provide stronger evidence, such as clearer biochemical enrichment of Rp1 in the analyzed fraction, focused classification to test RNA heterogeneity, or at minimum a more explicit acknowledgment that the RNA model is provisional in the peripheral/less-resolved regions.*

**Authors’ response:** We thanks the reviewer for this comment and fully agree with the observations made by the reviewer.

We are certain that both Rp1 and Rp2 are present in the purified TenpIN complex and can rescue TenpN induced growth inhibition in bacteria as shown in the results provided in the manuscript. We have also shown that TenpN can assemble with both Rp1 and Rp2 *in vitro*. Therefore, as reviewer has pointed out, we believe that the purified complex contain both Rp1 and Rp2 containing complexes.

However, we consistently got slightly better fit for Rp1 model in in the cryoEM density and therefore used Rp1 for building the model for the representational purposes in the manuscript. We have now lessoned our claim, wherever possible, and clearly mentioned the possibility of Rp2 complexes in the purified sample. **Please see the updated text on Page 9, Para 3.**

Moreover, due to the limitation of the resolution, we have limited our discussion, on details of RNA in the model, to only the backbone description without overstating the facts. We have only described protein TenpN putative active site residues based on the structure, which have been also experimentally validated in the revised manuscript. We have added these new results in the revised manuscript. **Please see the new results on Page 10-11 and new Figure 7b-d.**

***2.*** *The manuscript repeatedly refers to a “potential/plausible active site” and infers catalytic similarity to ToxN/CptN based largely on structural comparison and residue equivalence. However, no mutagenesis or direct RNase assay is presented to validate the proposed catalytic residues in TenpN. This is especially important because the discussion highlights specific residues, including Ser56, Arg55, and a unique His57, which further weakens confidence in this section. At present, the manuscript supports a putative active-site assignment, not a mechanistic conclusion. The authors should either add functional validation or substantially temper this section.*

**Authors’ response:** We thank the reviewer for this comment.

We have now validated a set of putative active site residues by mutagenesis approach. Based on the comparison with the ToxN and CptN, we had reported that residues Pro53, Leu54, Arg55, and Ser56 are near the active site and hence postulated them to be the possible putative active site residues in TenpN. To validate this, for this revision, we have made the alanine substitution of these residues and created single-point alanine mutants P53A, L54A, R55A, and S56A of TenpN.

We used bacterial growth assays to check the effect of TenpN mutants on *E. coli growth.* In the liquid LB medium, we observed that mutants had effect on the *E. coli* growth to different extent. P53A and L54A mutations in TenpN lead to the growth inhibition comparable to the WT TenpN, suggesting that L54A mutation does not lead to the inactivation of the toxin. However, in case of R55A and S56A we observed rescue of growth inhibition. We also repeated these experiments on solid LB agar medium, that also corroborated the results obtained in the liquid LB media. The growth inhibition caused by WT, P53A, L54A, and H57A mutants of TenpN were rescued upon co-expression of antitoxin ToxI.

Therefore, taken together, these results show that residues R55 and S56 play key role in the activity of TenpN in bacteria. Comparing their proximity to the potential cleavage site and comparison with the ToxN and CptN structures, we propose that these residue are likely going to be the active site residues in TenpN.

We have now added these results in the revised manuscript. **Please see the new results on Page 10-11 and new Figure 7b-d.**

*3. The authors show that both Rp1 and Rp2 rescue growth inhibition and that both can form higher-molecular-weight complexes with purified toxin by analytical SEC. However, the manuscript currently implies broader mechanistic insight into why this system uses two non-identical repeats, while the data mainly establish that both repeats are functional. Unless additional comparative biochemical evidence is provided—for example regarding repeat-specific processing, binding stability, or assembly behavior—the discussion should more clearly distinguish demonstrated findings from mechanistic speculation.*

**Authors’ response:** We thank the reviewer for this comment and agree that the narrative was giving an impression that two non-identical repeats have functional implications. However, as reviewer has noted, the evidences suggest that both the repeats are able to rescue the toxin induced growth inhibition to a similar extent.

For this revision, we have performed additional experiments to check the relative thermal stabilities of Rp1 and Rp1 using CD spectroscopy. The results again showed that both Rp1 and Rp2 form folded structures of similar thermal stabilities. We have added these results in the main manuscript**. Please see text on Page 7-8 and new Supplementary Figure S3.**

We have worked on the manuscript and in the revised manuscript presented the results accordingly, emphasizing that both the repeats are functional repeats and form complex with TenpN

***Part III – Minor Issues: Editorial and Data Presentation Modifications***

*Please use this section for editorial suggestions as well as relatively minor modifications of existing data that would enhance clarity.*

*Reviewer #1: Table S1 summarizes cryo-EM data collection and processing parameters but should also include details of model building and refinement.*

**Authors’ response:** Thanks for brining this to our notice. We have added the model building and refinement statistics in the **updated Supplementary Table T2**.

*Reviewer #2: 1. The methods need additional clarity in several places. For example, the text states induction with 100 mM IPTG, which is almost certainly incorrect and likely should be 100 µM or 1 mM. This should be verified carefully because as written it is implausible.*

**Authors’ response:** We thank the reviewer for pointing out this mistake. It is indeed 1 mM IPTG. This has now been corrected in the manuscript.

*2. The authors should ensure all placeholder citations are resolved. The “(REF)” in the active-site discussion is unacceptable in a submitted manuscript.*

**Authors’ response:** Thanks for bringing this to our notice. We have provided reference at all the places now.

*3. The authors should provide stronger figure support for the cryo-EM model quality, especially for the RNA. Given the modest resolution and partial RNA traceability, well-annotated local density panels are important to justify the model.*

**Authors’ response:** We have provided figures 5E-F and now **additional Supplementary Figures S8 and S9**. This figure clearly depicts the density of the RNA and fitted model to the density.

*4. The nomenclature and capitalization should be standardized throughout: ToxIN, CptIN, TenpIN; toxin/antitoxin names; figure panel references (“Figure 2C” vs “Fig. 2c”); and use of italics for gene names versus protein names.*

**Authors’ response:** We have corrected these errors (and like these errors) throughout the manuscript text.

*5. The keyword list contains a typo: “peudoknot” should be “pseudoknot.”*

**Authors’ response:** Corrected.

*6. The manuscript contains many grammatical and stylistic errors that require comprehensive language editing before publication.*

**Authors’ response:** Thanks for bring this to our notice. We have corrected the manuscript for all the grammatical and stylistic errors.

*7. Some structural descriptions are potentially inconsistent. For example, the text states “five β-strands (strands β1 to β4),” which numerically does not match. This likely needs correction in the fold description.*

**Authors’ response:** Thanks for bring this to our notice. It should indeed be strands β1 to β5. We have corrected this in the manuscript.

*8. There is also a clear typo in the CFU section (“Rp1, or Rp1” instead of “Rp1, or Rp2”), and the manuscript contains numerous grammatical errors throughout. These issues reduce confidence and should be addressed carefully.*

**Authors’ response:** Thanks for bring this error to our notice. We have now corrected all these errors it in the manuscript.

*9. The manuscript is categorized under “Gram Positive Bacteria,” while the studied system is described as common in Enterobacteriaceae including Salmonella, Shigella, Klebsiella, and E. coli, which are Gram-negative organisms. This should be checked at submission metadata level.*

**Authors’ response:** Thanks for point this out. We have categorized the manuscript now under Gram-negative category at the submission site.
