# Supplementary material for: Cryo-EM reveals the quaternary architecture of TenpIN type III toxin-antitoxin RNP complex common to pathogenic bacteria
Source: PLoS Pathog. 2026 Oct 6;22(10):e1014690. doi: 10.1371/journal.ppat.1014690 (PMC13682666; doi:10.1371/journal.ppat.1014690)
Supplement: Supplementary file 5 [file ppat.1014690.s013.docx]

**Graphical abstract**
