# Supplementary material for: Cryo-EM reveals the quaternary architecture of TenpIN type III toxin-antitoxin RNP complex common to pathogenic bacteria
Source: PLoS Pathog. 2026 Oct 6;22(10):e1014690. doi: 10.1371/journal.ppat.1014690 (PMC13682666; doi:10.1371/journal.ppat.1014690)
Supplement: Peer review history

# Peer review: Decision Letter 0

**DOI:** 10.1371/journal.ppat.1014690.r001

**Authors:** Michael Otto, Gongyi Zhang

14 Apr 2026

PPATHOGENS-D-26-00539

Cryo-EM reveals the quaternary architecture of TenpIN type III toxin-antitoxin RNP complex common to pathogenic bacteria

PLOS Pathogens

Dear Dr. Singh,

Thank you for submitting your manuscript to PLOS Pathogens. After careful consideration, we feel that it has merit but does not fully meet PLOS Pathogens's publication criteria as it currently stands. Therefore, we invite you to submit a revised version of the manuscript that addresses the points raised during the review process.

Please submit your revised manuscript by Jun 13 2026 11:59PM. If you will need more time than this to complete your revisions, please reply to this message or contact the journal office at plospathogens@plos.org. When you're ready to submit your revision, log on to https://www.editorialmanager.com/ppathogens/ and select the 'Submissions Needing Revision' folder to locate your manuscript file.

Please include the following items when submitting your revised manuscript:

* A letter that responds to each point raised by the editor and reviewer(s). You should upload this letter as a separate file labeled 'Response to Reviewers'. This file does not need to include responses to any formatting updates and technical items listed in the 'Journal Requirements' section below.

* A marked-up copy of your manuscript that highlights changes made to the original version. You should upload this as a separate file labeled 'Revised Manuscript with Track Changes'.

* An unmarked version of your revised paper without tracked changes. You should upload this as a separate file labeled 'Manuscript'.

If you would like to make changes to your financial disclosure, competing interests statement, or data availability statement, please make these updates within the submission form at the time of resubmission. Guidelines for resubmitting your figure files are available below the reviewer comments at the end of this letter.

We look forward to receiving your revised manuscript.

Kind regards,

Gongyi Zhang

Academic Editor

PLOS Pathogens

Michael Otto

Section Editor

PLOS Pathogens

Sumita Bhaduri-McIntosh

Editor-in-Chief

PLOS Pathogens

orcid.org/0000-0003-2946-9497

Michael Malim

Editor-in-Chief

PLOS Pathogens

orcid.org/0000-0002-7699-2064

**Journal Requirements:**

1) Please ensure that the CRediT author contributions listed for every co-author are completed accurately and in full.

At this stage, the following Authors/Authors require contributions: Kavyashree Nadig, Surekha Padmanaban, Salik Noor, Sankaran Sandhya, Parthasarathy Manikandan, Somnath Dutta, and Mahavir Singh. Please ensure that the full contributions of each author are acknowledged in the "Add/Edit/Remove Authors" section of our submission form.

The list of CRediT author contributions may be found here: https://journals.plos.org/plospathogens/s/authorship#loc-author-contributions

2) Please provide an Author Summary. This should appear in your manuscript between the Abstract (if applicable) and the Introduction, and should be 150-200 words long. The aim should be to make your findings accessible to a wide audience that includes both scientists and non-scientists. Sample summaries can be found on our website under Submission Guidelines:

https://journals.plos.org/plospathogens/s/submission-guidelines#loc-parts-of-a-submission

3) We do not publish any copyright or trademark symbols that usually accompany proprietary names, eg ©,  ®, or TM  (e.g. next to drug or reagent names). Therefore please remove all instances of trademark/copyright symbols throughout the text, including:

- ® on page: 14

- TM on page: 13.

4) Please upload all main figures as separate Figure files in .tif or .eps format. For more information about how to convert and format your figure files please see our guidelines:

https://journals.plos.org/plospathogens/s/figures

5) We have noticed that you have uploaded Supporting Information files, but you have not included a list of legends. Please add a full list of legends for your Supporting Information files after the references list.

6) Thank you for stating that " The EM maps and the coordinates of the TenpIN complex have been deposited to Electron Microscopy Data Bank (EMDB) and Protein Data Bank (PDB) under the accession codes: EMD-64795 and 9V7X respectively". Please note that, though access restrictions are acceptable now, your entire minimal dataset will need to be made freely accessible if your manuscript is accepted for publication. This policy applies to all data except where public deposition would breach compliance with the protocol approved by your research ethics board. If you are unable to adhere to our open data policy, please kindly revise your statement to explain your reasoning and we will seek the editor's input on an exemption.

7) Please amend your detailed Financial Disclosure statement. This is published with the article. It must therefore be completed in full sentences and contain the exact wording you wish to be published.

1) State what role the funders took in the study. If the funders had no role in your study, please state: "The funders had no role in study design, data collection and analysis, decision to publish, or preparation of the manuscript."

8) Please ensure that the funders and grant numbers match between the Financial Disclosure field and the Funding Information tab in your submission form. Note that the funders must be provided in the same order in both places as well.

Note: If the reviewer comments include a recommendation to cite specific previously published works, please review and evaluate these publications to determine whether they are relevant and should be cited. There is no requirement to cite these works unless the editor has indicated otherwise.

**Reviewers' Comments:**

Reviewer's Responses to Questions

**Part I - Summary**

Please use this section to discuss strengths/weaknesses of study, novelty/significance, general execution and scholarship.

Reviewer #1: In this manuscript, the authors characterize a type III toxin–antitoxin system, TenpIN, which is reported to be universally present in the Enterobacteriaceae family. Bacterial growth and rescue assays were performed to demonstrate the toxicity of the TenpN protein and the antitoxin activity of RNA repeats. The structure of the TenpN–RNA complex was determined at ~4 Å resolution using cryo-EM, revealing a heterotetrameric arrangement. However, given the relatively low resolution, detailed protein/RNA interactions cannot be resolved, which significantly limits the overall scientific impact of the study.

Reviewer #2: This manuscript reports the first structural characterization of a TenpIN type III toxin–antitoxin complex and therefore addresses a genuine gap in the field. The combination of functional assays in E. coli, biochemical purification, and cryo-EM reconstruction supports the central claim that the selected TenpIN system forms a 2:2 protein:RNA assembly and that both non-identical RNA repeats are functionally competent antitoxins. The work is potentially publishable, and the structural finding is of clear interest. However, in its current form, the manuscript overstates several conclusions, leaves key mechanistic points insufficiently supported, and contains a number of textual inconsistencies, methodological ambiguities, and typographical problems that need careful revision. In my view, the manuscript contains a structurally interesting dataset and could become suitable for publication after substantial revision, but several of the current mechanistic and structural interpretations need to be either experimentally strengthened or more carefully delimited.

**********

**Part II – Major Issues: Key Experiments Required for Acceptance**

Please use this section to detail the key new experiments or modifications of existing experiments that should be absolutelyrequired to validate study conclusions.

Generally, there should be no more than 3 such required experiments or major modifications for a "Major Revision" recommendation. If more than 3 experiments are necessary to validate the study conclusions, then you are encouraged to recommend "Reject".

Reviewer #1: 1. No cryo-EM density map has been provided to the reviewers, making it difficult to assess the quality of the reconstruction and the agreement between the model and the map.

2. The authors state that the “left shoulder” of the SEC peak was used for cryo-EM analysis because it behaves markedly differently from the “central” and “right” portions of the peak. However, no details are provided regarding which elution fractions correspond to each region. Additionally, based on Supplementary Figure S3, only the TenpN/Rp2 complex appears to exhibit a left shoulder. Was this the sample used for the cryo-EM study? If so, why was Rp1 used for model building instead of Rp2? Furthermore, on page 8, the authors state that “cryo-EM sample preparation involved collecting particles from a specific fraction after SEC (from the right shoulder of the elution peak),” which is inconsistent with the earlier description and therefore confusing.

Reviewer #2: 1. A central issue is that the final structural interpretation relies on fitting Rp1 into both RNA densities, even though the authors state that the purified complex contains both Rp1 and Rp2. Their rationale is indirect: Rp1 fits better, Rp2 is shorter by three nucleotides, Rp2 rescue appears somewhat weaker in spot assays, and the authors speculate that the cryo-EM fraction may be enriched for Rp1-containing complexes. This is plausible, but still not definitive. As written, the manuscript risks giving the impression that the structure represents the biologically dominant state, while the data seem more consistent with a model of one plausible repeat-derived density assignment rather than an unambiguous Rp1-only structure. The claims should therefore be softened unless the authors can provide stronger evidence, such as clearer biochemical enrichment of Rp1 in the analyzed fraction, focused classification to test RNA heterogeneity, or at minimum a more explicit acknowledgment that the RNA model is provisional in the peripheral/less-resolved regions.

2. The manuscript repeatedly refers to a “potential/plausible active site” and infers catalytic similarity to ToxN/CptN based largely on structural comparison and residue equivalence. However, no mutagenesis or direct RNase assay is presented to validate the proposed catalytic residues in TenpN. This is especially important because the discussion highlights specific residues, including Ser56, Arg55, and a unique His57, which further weakens confidence in this section. At present, the manuscript supports a putative active-site assignment, not a mechanistic conclusion. The authors should either add functional validation or substantially temper this section.

3. The authors show that both Rp1 and Rp2 rescue growth inhibition and that both can form higher-molecular-weight complexes with purified toxin by analytical SEC. However, the manuscript currently implies broader mechanistic insight into why this system uses two non-identical repeats, while the data mainly establish that both repeats are functional. Unless additional comparative biochemical evidence is provided—for example regarding repeat-specific processing, binding stability, or assembly behavior—the discussion should more clearly distinguish demonstrated findings from mechanistic speculation.

**********

**Part III – Minor Issues: Editorial and Data Presentation Modifications**

Please use this section for editorial suggestions as well as relatively minor modifications of existing data that would enhance clarity.

Reviewer #1: Table S1 summarizes cryo-EM data collection and processing parameters but should also include details of model building and refinement.

Reviewer #2: 1. The methods need additional clarity in several places. For example, the text states induction with 100 mM IPTG, which is almost certainly incorrect and likely should be 100 µM or 1 mM. This should be verified carefully because as written it is implausible.

2. The authors should ensure all placeholder citations are resolved. The “(REF)” in the active-site discussion is unacceptable in a submitted manuscript.

3. The authors should provide stronger figure support for the cryo-EM model quality, especially for the RNA. Given the modest resolution and partial RNA traceability, well-annotated local density panels are important to justify the model.

4. The nomenclature and capitalization should be standardized throughout: ToxIN, CptIN, TenpIN; toxin/antitoxin names; figure panel references (“Figure 2C” vs “Fig. 2c”); and use of italics for gene names versus protein names.

5. The keyword list contains a typo: “peudoknot” should be “pseudoknot.”

6. The manuscript contains many grammatical and stylistic errors that require comprehensive language editing before publication.

7. Some structural descriptions are potentially inconsistent. For example, the text states “five β-strands (strands β1 to β4),” which numerically does not match. This likely needs correction in the fold description.

8. There is also a clear typo in the CFU section (“Rp1, or Rp1” instead of “Rp1, or Rp2”), and the manuscript contains numerous grammatical errors throughout. These issues reduce confidence and should be addressed carefully.

9. The manuscript is categorized under “Gram Positive Bacteria,” while the studied system is described as common in Enterobacteriaceae including Salmonella, Shigella, Klebsiella, and E. coli, which are Gram-negative organisms. This should be checked at submission metadata level.

**********

PLOS authors have the option to publish the peer review history of their article (what does this mean?). If published, this will include your full peer review and any attached files.

If you choose “no”, your identity will remain anonymous but your review may still be made public.

**Do you want your identity to be public for this peer review?** For information about this choice, including consent withdrawal, please see our Privacy Policy.

Reviewer #1: No

Reviewer #2: No

[NOTE: If reviewer comments were submitted as an attachment file, they will be attached to this email and accessible via the submission site. Please log into your account, locate the manuscript record, and check for the action link "View Attachments". If this link does not appear, there are no attachment files.]

**Figure resubmission:**

While revising your submission, we strongly recommend that you use PLOS’s NAAS tool (https://ngplosjournals.pagemajik.ai/artanalysis) to test your figure files. NAAS can convert your figure files to the TIFF file type and meet basic requirements (such as print size, resolution), or provide you with a report on issues that do not meet our requirements and that NAAS cannot fix.

After uploading your figures to PLOS’s NAAS tool - https://ngplosjournals.pagemajik.ai/artanalysis, NAAS will process the files provided and display the results in the "Uploaded Files" section of the page as the processing is complete. If the uploaded figures meet our requirements (or NAAS is able to fix the files to meet our requirements), the figure will be marked as "fixed" above. If NAAS is unable to fix the files, a red "failed" label will appear above. When NAAS has confirmed that the figure files meet our requirements, please download the file via the download option, and include these NAAS processed figure files when submitting your revised manuscript.

**Reproducibility:**

To enhance the reproducibility of your results, we recommend that authors of applicable studies deposit laboratory protocols in protocols.io, where a protocol can be assigned its own identifier (DOI) such that it can be cited independently in the future. Additionally, PLOS ONE offers an option to publish peer-reviewed clinical study protocols. Read more information on sharing protocols at https://plos.org/protocols?utm_medium=editorial-email&utm_source=authorletters&utm_campaign=protocols

# Author comment: Author response to Decision Letter 1

**DOI:** 10.1371/journal.ppat.1014690.r002

20 Aug 2026

Attachment Submitted filename: Response_to_reviewers_17aug2026.docx

# Editor report: Decision Letter 1

**DOI:** 10.1371/journal.ppat.1014690.r003

**Authors:** Michael Otto, Gongyi Zhang

29 Aug 2026

Dear Dr. Singh,

We are pleased to inform that your manuscript, "Cryo-EM reveals the quaternary architecture of TenpIN type III toxin-antitoxin RNP complex common to pathogenic bacteria", has been editorially accepted for publication at PLOS Pathogens.

Before your manuscript can be formally accepted and sent to production, you will need to complete our formatting changes, which you will receive by email within a week. Please note that your manuscript will not be scheduled for publication until you have made the required changes.

IMPORTANT NOTES

(1) Copyediting and Proofreading: The corresponding author will receive a typeset proof for review, to ensure errors have not been introduced during production. Please review the PDF proof of your manuscript carefully, as this is the last chance to correct any errors. Please note that major changes, or those which affect the scientific understanding of the work, will likely cause delays to the publication date of your manuscript.

(2) Appropriate Figure Files: Please remove all name and figure # text from your figure files. Please also take this time to check that your figures are of high resolution, which will improve the readbility of your figures and help expedite your manuscript's publication. Please note that figures must have been originally created at 300dpi or higher. Do not manually increase the resolution of your files. For instructions on how to properly obtain high quality images, please review our Figure Guidelines, with examples at: http://journals.plos.org/plospathogens/s/figures.

We encourage you to use PACE (http://pace.apexcovantage.com/), a figure-checking tool which helps authors ensure all figures meet PLOS requirements so that figures can publish in the highest quality. PACE is a digital diagnostic and conversion tool for figure files. It will provide information about any failed check(s) and, if able, will automatically convert the figure into an acceptable file that passes quality checks.

To use PACE, you must first register as a user. Then, login and navigate to the UPLOAD tab, where you will find detailed instructions on how to use the tool.

(3) Striking Image: Please upload a striking still image to accompany your article if one is available (you can include a new image or an existing one from within your manuscript). Should your paper be accepted, this image will be considered for our monthly issue image and may also appear on our website to feature your article. Please upload this as a separate file, selecting "striking image" as the file type upon upload. Please also include a separate "Other" file with a caption, including credits and any potential copyright information. Please do not include the caption in the main article file. If your image is from someone other than yourself, please ensure that the artist has read and agreed to the terms and conditions of the Creative Commons Attribution License at http://journals.plos.org/plospathogens/s/content-license. Please note that PLOS cannot publish copyrighted images.

(4) Press Release or Related Media: If you or your institution will be preparing press materials for this manuscript, please inform our press team in advance at plospathogens@plos.org as soon as possible. We ask that you contact us within one week to plan ahead of our fast Production schedule. If you need to know your paper's publication date for related media purposes, you must coordinate with our press team, and your manuscript will remain under a strict press embargo until the publication date and time.

(5) Update your Profile Information: Now that your manuscript has been provisionally accepted, please log into Editorial Manager and update your profile, if needed. Go to http://www.editorialmanager.com/ppathogens, log in, and click on the "Update My Information" link at the top of the page. Please update your user information to ensure an efficient production and billing process.

(6) LaTeX users only: Our staff will ask you to upload a TEX file in addition to the PDF before the paper can be sent to typesetting, so please carefully review our Latex Guidelines http://journals.plos.org/plospathogens/s/latex in the meantime.

Thank you again for supporting Open Access publishing; we are looking forward to publishing your work in PLOS Pathogens.

Best regards,

Gongyi Zhang

Academic Editor

PLOS Pathogens

Michael Otto

Section Editor

PLOS Pathogens

Dominique Soldati-Favre

Editor-in-Chief

PLOS Pathogens

***********************************************************

# Editor report: Acceptance letter

**DOI:** 10.1371/journal.ppat.1014690.r004

**Authors:** Michael Otto, Gongyi Zhang

Dear Dr. Singh,

We are delighted to inform you that your manuscript, "Cryo-EM reveals the quaternary architecture of TenpIN type III toxin-antitoxin RNP complex common to pathogenic bacteria," has been formally accepted for publication in PLOS Pathogens.

We have now passed your article onto the PLOS Production Department who will complete the rest of the pre-publication process. All authors will receive a confirmation email upon publication.

Thank you again for supporting open-access publishing; we are looking forward to publishing your work in PLOS Pathogens.

Best regards,

Dominique Soldati-Favre

Editor-in-Chief

PLOS Pathogens
